# Combined mitoxantrone and anti-TGFβ treatment with PD-1 blockade enhances antitumor immunity by remodelling the tumor immune landscape in neuroblastoma

**DOI:** 10.1186/s13046-022-02525-9

**Published:** 2022-11-17

**Authors:** Valeria Lucarini, Ombretta Melaiu, Silvia D’Amico, Fabio Pastorino, Patrizia Tempora, Marco Scarsella, Marco Pezzullo, Adele De Ninno, Valentina D’Oria, Michele Cilli, Laura Emionite, Paola Infante, Lucia Di Marcotullio, Maria Antonietta De Ioris, Giovanni Barillari, Rita Alaggio, Luca Businaro, Mirco Ponzoni, Franco Locatelli, Doriana Fruci

**Affiliations:** 1 Department of Paediatric Haematology/Oncology and of Cell and Gene Therapy, Bambino Gesù Children’s Hospital, IRCCS, 00146 Rome, Italy; 2 Department of Clinical Sciences and Translational Medicine, University of Rome “Tor Vergata”, Rome, Italy; 3 Laboratory of Experimental Therapies in Oncology, IRCCS Istituto Giannina Gaslini, 16147 Genoa, Italy; 4 Flow Cytometry Core Facility, Bambino Gesù Children’s Hospital, IRCCS, 00146 Rome, Italy; 5 Research Laboratories, Bambino Gesù Children’s Hospital, IRCCS, 00146 Rome, Italy; 6 CNR Institute for Photonics and Nanotechnology, Rome, 00156 Italy; 7 Confocal Microscopy Core Facility, Research Center, Bambino Gesù Children’s Hospital, IRCCS, 00146 Rome, Italy; 8 IRCCS Ospedale Policlinico San Martino, Animal Facility, 16132 Genoa, Italy; 9 Department of Molecular Medicine, University La Sapienza, 00161 Rome, Italy; 10 Pathology Unit, Bambino Gesù Children’s Hospital, IRCCS, 00146 Rome, Italy; 11 Department of Life Sciences and Public Health, Catholic University of the Sacred Heart, 00168 Rome, Italy

**Keywords:** Neuroblastoma, Immunotherapy, Tumor Microenvironment, Immunomodulation, Drug Evaluation

## Abstract

**Background:**

Poor infiltration of functioning T cells renders tumors unresponsive to checkpoint-blocking immunotherapies. Here, we identified a combinatorial in situ immunomodulation strategy based on the administration of selected immunogenic drugs and immunotherapy to sensitize poorly T-cell-infiltrated neuroblastoma (NB) to the host antitumor immune response.

**Methods:**

975A2 and 9464D NB cell lines derived from spontaneous tumors of TH-MYCN transgenic mice were employed to study drug combinations able of enhancing the antitumor immune response using in vivo and ex vivo approaches. Migration of immune cells towards drug-treated murine-derived organotypic tumor spheroids (MDOTS) were assessed by microfluidic devices. Activation status of immune cells co-cultured with drug-treated MDOTS was evaluated by flow cytometry analysis. The effect of drug treatment on the immune content of subcutaneous or orthotopic tumors was comprehensively analyzed by flow-cytometry, immunohistochemistry and multiplex immunofluorescence. The chemokine array assay was used to detect soluble factors released into the tumor microenvironment. Patient-derived organotypic tumor spheroids (PDOTS) were generated from human NB specimens. Migration and activation status of autologous immune cells to drug-treated PDOTS were performed.

**Results:**

We found that treatment with low-doses of mitoxantrone (MTX) recalled immune cells and promoted CD8^+^ T and NK cell activation in MDOTS when combined with TGFβ and PD-1 blockade. This combined immunotherapy strategy curbed NB growth resulting in the enrichment of a variety of both lymphoid and myeloid immune cells, especially intratumoral dendritic cells (DC) and IFNγ- and granzyme B-expressing CD8^+^ T cells and NK cells. A concomitant production of inflammatory chemokines involved in remodelling the tumor immune landscape was also detected. Interestingly, this treatment induced immune cell recruitment against PDOTS and activation of CD8^+^ T cells and NK cells.

**Conclusions:**

Combined treatment with low-dose of MTX and anti-TGFβ treatment with PD-1 blockade improves antitumor immunity by remodelling the tumor immune landscape and overcoming the immunosuppressive microenvironment of aggressive NB.

**Supplementary Information:**

The online version contains supplementary material available at 10.1186/s13046-022-02525-9.

## Background

Immune checkpoint inhibitors (ICI) have shown impressive clinical results against a variety of highly aggressive tumors, although durable benefits have only been observed in a limited fraction of patients [1–3]. The main reason for this non-response is the lack of functional tumor-infiltrating T cells [4]. Given the great potential of ICI, it is of paramount importance to improve their efficacy by populating less infiltrated tumors with functional immune cells.

Compelling evidence shows that in order to function, T cells required a favourable immune microenvironment in which other immune actors play a crucial role [5]. Indeed, tumors that respond to immunotherapy are enriched not only in T cells, but also in other immune cell populations, including conventional type 1 dendritic cells (DC1) and natural killer (NK) cells, which, by interacting with each other, create an environment suitable for the priming and expansion of tumor-specific T cells [5]. Consistently, similarly to CD8^+^ T cells, intratumoral DC1 and NK cells have also been associated with a strong antitumor response and favourable clinical outcome in various tumors [6]. The development of novel approaches that can restore the function of the entire immune cycle is therefore of crucial importance to increase the number of patients who can benefit from immunotherapy. In this regard, several chemotherapeutic treatments have been shown to recall immune cells in the tumor microenvironment (TME) through different mechanisms [7]. For examples, some chemotherapeutics such as doxorubicin (DX), mitoxantrone (MTX), oxaliplatin (OXP), and cyclophosphamide, when administrated at low doses are able to induce immunogenic cell death (ICD) by promoting DC activation, antigen presentation and priming of tumor-specific CD8^+^ T cells [7–10].

However, an increase in intratumoral immune cell infiltrate may not be conclusive because of the multiple mechanisms adopted by tumors to prevent patients’ immune system from targeting and eliminating their own tumor cells [3]. Recently, transcriptional profiling of melanoma and metastatic urothelial carcinoma patients unresponsive to anti-PD-1 therapy revealed an enrichment in pathways associated to TGFβ [11, 12], a cytokine produced by the TME that reduces immune cell recall within the tumor and inhibits the function of both T cells and NK cells [13]. Consistently, recent use of TGFβ-blocking antibodies has been shown to overcome resistance to anti-PD-1 therapy by reactivating the antitumor immune response in colorectal cancer and melanomas [12, 14].

Approaches aimed at improving the effectiveness of treatments in a increasing number of patients, in combination with therapies that enhance the recruitment of innate and adaptive immune cells, could prove valuable for currently intractable tumors, such as neuroblastoma (NB), a paediatric cancer of the peripheral sympathetic system that accounts for 15% of all childhood cancer-related deaths [15–19]. Despite advanced and intensive multidisciplinary therapeutic approaches based on induction chemotherapy, myeloablative chemotherapy, surgery, radiotherapy and treatment of minimal residual disease, mortality of patients with high-risk NB remains significant, and those who survive experience severe long-term side effects. Therefore, it is important to develop more effective and tolerated therapeutic approaches.

Extensive pathological studies of over than 100 cases of primary and metastatic human NBs examined by immunohistochemistry (IHC) revealed the importance of the immune content in the NB microenvironment [20–22]. NB highly infiltrated by T cells are also enriched with DC and NK cells, and the abundance of these immune cell populations is positively correlated with a favourable clinical outcome [20–22]. The importance of the intratumoral immune context in NB is further strengthened by the identification of intratumoral gene signatures specific to these immune cell populations that correlate with PD-1 and PD-L1 expression levels [22].

Similarly to other tumors, NB exploits a variety of immune evasion strategies including expression of immune checkpoint molecules, induction of immunosuppressive cells, as well as secretion of immunomodulatory mediators, including TGFβ [23–26]. The development of novel multiple combined immunotherapeutic protocols to recruit innate and adaptive immune cells and convert the immunosuppressive environment to an immunostimulatory one may be effective in the treatment of NB.

In this work, we employ a combinatorial in situ immunomodulation strategy based on the administration of i) low dose of chemotherapy to promote ICD and mobilize immune cells into the TME, ii) anti-TGFβ to overcome immunosuppression in the TME, iii) and anti-PD-1 to restore the function of tumor-infiltrating immune cells. Using ex vivo approaches with murine- and patients-derived NB tissues, as well mouse models of NB grown subcutaneously or orthotopically in the adrenal gland, we identify a novel combination therapy that recruits a variety of innate and adaptive immune cells into the TME, and significantly reduces the growth of this aggressive tumor.

## Methods

### Mice, cell lines and reagents

Six to 8-week-old female C57BL/6 black and albino mice (Charles River Laboratories) were housed under pathogen-free conditions in the Plaisant (Rome, Italy) and in Ospedale Policlinico San Martino (Genova, Italy) animal facilities, respectively. In vivo experiments were performed in accordance with the 3Rs policy and reviewed and approved by the Italian Ministry of Health (authorization n. 755/2019-PR).

Transgenic NB cell lines 9464D and 975A2 were derived from spontaneous tumors arising in TH-MYCN transgenic mice on a C57BL/6 background [27] and kindly gift by Dr. Crystal Mackall (Stanford University, CA). Tumor cells were grown under standard conditions (RPMI with 10% FCS with Pen/Strep/Glut at 37 °C and 5% CO_2_) on tissue-culture treated plastic plates, splitted every other day prior to injection into mice, passaged no more than four times since thawing and routinely tested for the absence of mycoplasma.

DX hydrochloride, MTX dihydrochloride and OXP were from Sigma-Aldrich. Cisplatin (CDDP, Accord Healthcare Limited), vincristine (VINC, Pfizer) and irinotecan (IRI, Campo, Pfizer) were kindly provided by the pharmacy of the Children’s Hospital Bambino Gesù (Rome, Italy).

### Lentiviral infection

9464D cells stably expressing luciferase were obtained by infection with CMV Lentivector Plasmid expressing Luciferase-EF1a-copGFP (BLIV511PA-1, System Biosciences), as previously described [21]. GFP-positive cells were sorted by a BD FACS Aria II and used for in vivo experiments.

### Flow-cytometry

All antibodies were purchased from BD Biosciences, eBioscience, Biolegend and R&D system (listed in Supplementary Table S1). For surface staining, cells were stained with fluorescent labelled antibodies in PBS with 2% FCS for 30 minutes on ice. Viability was assessed by staining with fixable Live/Dead Zombie (Biolegend) or DAPI. For intracellular staining, cells were seeded (1 × 10^6^ cells per well) in 96-well U-bottomed plates and stained with antibodies against surface markers, fixed with 2% PFA for 10 minutes at 25 °C, permeabilized with 0.2% Saponin and then stained with anti-FOXP3, anti-IFNγ and anti-granzyme B using Fixation/Permeabilization Concentrate and Diluent kit (eBioscience). Samples were analyzed on a BD Fortessa flow cytometer and FlowJo software (Treestar, version 10.7.2).

### MDOTS and PDOTS generation, drug-treatment and co-culture experiments

Murine-derived organotypic tumor spheroids (MDOTS) were obtained from tumor masses as previously described [28]. Briefly, tumors were mechanically dissected with sterile forceps and scissors, minced against a 70-μm pore filter with a syringe plunger, and washed in 5 ml of RPMI medium (1500 rpm, 5 minutes). The cell pellet was re-suspended in 2–5 ml RPMI medium, depending on the amount of sample, and passed through a 40-μm pore filter. The cell suspension was seeded into ultra-low attachment (ULA) plates at a density of 2000 cells per well in 96-well plates with 200 μl medium, 100,000 cells per well of 24-well plate with 750 μl medium, 300,000–500,000 cells per well of 6-well plate with 2 ml medium (Corning, Costar #3471, 3473, 3474).

Patient-derived organotypic tumor spheroids (PDOTS) were obtained from tumor samples of 7 patients with NB diagnosed between 2020 and 2021 at Bambino Gesù Children’s Hospital (Rome, Italy). Written informed parental consent was obtained for each patient in accordance with the Declaration of Helsinki. The study was approved by the institute’s Ethics Committee. Clinical and genetic information is given in Supplementary Table S2. Diagnosis and histology were performed according to the International Neuroblastoma Risk Group (INRG) staging system and the International Neuroblastoma Pathology Classification (INPC) [29, 30], respectively. MYCN status was assessed according to current guidelines [31]. PDOTS were obtained from tumor masses as previously described [28]. Briefly, human fresh tumor specimens were minced in a 10-cm dish using sterile forceps and scalpel. Minced tumors were re-suspended in NB PDOTS-medium (RPMI with 20% FCS, Pen/Strep/Glut 1X, Hepes 1X, NaPYR 1X, NEAA 1X and B27 1X at 37 °C and 5% CO_2_), strained on 70-μm filter and cultured in ULA tissue culture plates. MDOTS and PDOTS were treated overnight with the indicated drugs (2 μM DX, 2.5 μM OXP, 5 μM IRI for MDOTS, and 3 μM MTX, 5 μM anti-TGFβ and 5 μM anti-PD-1, for both MDOTS and PDOTS). The day after, MDOTS and PDOTS were washed to remove the drug-containing medium. Next, drug-treated and untreated MDOTS and PDOTS were co-cultured 24 hours with splenocytes derived from tumor-bearing C57BL/6 mice and autolougous human peripheral blood mononuclear cells (PBMC), respectively. The functional status of the immune cells was assessed by flow-cytometry using BD LSR Fortessa X20 with FACSDiva Software (BD Bioscences) and FlowJo software (version 10.7.2).

### Microfluidic device migration assay

To evaluate the immunomodulatory ability of drugs, we carried out experiments in microfluidic devices made of polydimethylsiloxane (PDMS), a biocompatible silicone elastomer, following a well-established replica moulding procedure [32, 33]. Prior to cell loading, the devices were sterilized under UV light for 30 minutes. MDOTS and PDOTS were re-suspended in Matrigel (2 mg/ml; BD Biosciences) and treated with drugs at the indicated concentrations. Drug-treated and untreated MDOTS and PDOTS were loaded into the side chambers of the devices (1 × 10^4^ cells in 3 μl), which are separated from the central one by microchannels, and incubated at 37 °C for 30 minutes to allow gel solidification. Subsequently, 1 × 10^6^ splenocytes derived from tumor-bearing C57BL/6 and autologous PBMC for MDOTS and PDOTS, respectively, were labelled with Cell Tracker Red (Invitrogen), re-suspended in complete RPMI medium and loaded into the central chamber of the device. The size of the microchannels allows splenocytes to migrate from the central chamber to the two lateral chambers, but not MDOTS/PDOTS to move into the central chamber. The reservoir chambers were filled with medium. Phase-contrast, visible and fluorescence microphotographs of the devices were taken with a LEICA DMi8 microscope by collecting photos at 24 hours after loading. The migration of splenocytes/PBMC towards treated and untreated MDOTS/PDOTS was assessed by counting the red-labeled cells in the two side chambers with the ImageJ software (http://imagej.nih.gov/ij). The extent of splenocytes/PBMC migration to treated versus untreated MDOTS/PDOTS was then analyzed in terms of fold change ± SD [32–34].

### Subcutaneous tumor model and therapeutic studies

9464D and 975A2 cells (1 × 10^6^) were inoculated subcutaneously into the flank of C57BL/6 mice. Survival of mice was monitored daily and tumor growth was measured twice weekly using a caliper. Mice were randomized into control and treatment groups (10 mice/group) at day 5, when the tumor volume reached 30–50 mm^3^. Drug treatment started at day 7 or 8. DX (2.9 mg/Kg) and OXP (2.5 mg/Kg) were injected intratumorally, whereas CDDP (0.25 mg/Kg), MTX (5.2 mg/kg), IRI (2.5 mg/Kg), VINC (0.5 mg/Kg), anti-PD-1 (clone RMP1–14, BE0146, BioXCell, 0.3 mg/mouse) and anti-TGFβ (clone 1D11.16.8, BP0057, BioXCell, 10 mg/Kg) were injected intraperitoneally. Control mice received an equivalent volume of PBS alone or isotype control antibody. Mice were sacrificed after 1 day or 7 or 12 days from the start of drug treatment for analysis of the tumor’s immune infiltrate. All experiments contained 5 to 10 mice per group and were performed at least 2 times, yielding similar results.

### Orthotopic tumor model and therapeutic studies

After anesthetization with a mixture of xylazine-ketamine (Xilor 2% Bio98 Srl, Milan, Italy) and Imalgene 1000 (Merial SpA, Italy), six-week-old C57BL/6 albino mice were subjected to laparotomy and inoculated with 0.7 × 10^6^ 9464D-luc cells in 10 μL culture medium, in the left adrenal gland capsule, as previously described [35]. Luc activity was confirmed by bio-luminescent imaging (BLI, Lumina-II, Caliper Life Sciences, Hopkinton, MA) after a 10-minute incubation with 150 μg/mL d-luciferin (Caliper Life Sciences) diluted in cell culture medium, as previously described [36]. BLI monitoring was used as the main criterion for determining the start of treatment. Mice body weight and general physical status were daily recorded. When any sign of discomfort or poor health arose (i.e., abdominal dilatation, dehydration, paraplegia, ≥20% weight loss) mice were anaesthetized with Xilor 2% and sacrificed by CO_2_ inhalation. When the tumor volume reached 1 × 10^7^ ROI measurement, the mice were randomized into control and treatment groups (6 mice/group). MTX (5.2 mg/kg), anti-PD-1 (0.3 mg/mouse) and anti-TGFβ (10 mg/Kg) were injected intraperitoneally. Control mice received an equivalent volume of PBS alone. Seven days after drug treatment, mice were sacrificed for analysis of the tumor immune infiltrate.

### Tumor dissection

Tumors and spleens were dissected from mice and total weight of removed tumor masses was determined. Tumors were cut into small fragments with scissors and then digested in medium containing 325 KU/ml DNAse I (Sigma) and 1 mg/ml Collagenase III (Worthington Biochemicals) per 30 minutes at room temperature in agitation followed by 0.1 M EDTA pH 7.2 for additional 5 minutes. Samples are then filtered through a 70 μm filter, spun down and re-suspended for staining.

### Immunofluorescence and immunohistochemistry

Immunofluorescence (IF) and IHC stainings were performed in 2 μm of formaldehyde-fixed paraffin embedded serial tissue sections following deparaffinization and antigen retrieval as previously described [21, 22]. For double IF staining of NK cells and granzyme B, slides were blocked for 1 hour with 1% BSA and 5% normal goat serum and then antibodies (Supplementary Table S1) were added consecutively as follow. Sections were firstly incubated with anti-granzyme B antibody overnight at 4 °C, followed by 1-hour incubation with Alexa Fluor 594 goat anti-rabbit IgG. Next, slides were incubated with anti-NK1.1 overnight at 4 °C, followed by 1-hour incubation with Alexa Fluor 488 goat anti-mouse IgG. After staining, slides were counterstained for 5 minutes with Hoechst (H3570, Invitrogen) and cover-slipped with 60% glycerol in PBS. Confocal microscopy imaging was performed by Leica TCS-SP8Xlaser-scanning confocal microscope (Leica Microsystems) equipped with tunable white light laser source, 405 nm diode laser, 3 (PMT) e 2(HyD) internal spectral detector channels. Sequential confocal images were acquired using a HC PLAPO 40× oil immersion objective (1.30 numerical aperture, Leica Microsystems) with a 1024 × 1024 image format, scan speed 400 Hz. The density of intratumoral NK cells was recorded by two blinded examiners as the number of positive cells per unit tissue surface area (mm^2^). The mean of the positive cells detected in 5 fields for each sample was used in the statistical analysis.

For calreticulin (CALR) staining, slides were blocked for endogenous peroxidase for 10 minutes with a peroxidase blocking solution (Dako), followed by 30 minutes with 5% PBS/BSA, and then incubated (overnight at 4 °C) with anti-CARL primary antibody (Supplementary Table S1). This step was followed by incubation with secondary antibody coupled with peroxidase (Dako) for 20 minutes. Bound peroxidase was detected with diaminobenzidine solution and EnVision FLEX Substrate buffer containing peroxide (Dako). Tissue sections were counterstained with EnVision FLEX hematoxylin (Dako). Iso-type-matched mouse mAbs were used as negative controls. Stained slides were analyzed using an image analysis workstation (Nikon Eclipse E600), scanned using the NanoZoomer S60 Digital slide scanner C13210–01 (Hamamatsu Photonics) and viewed with Hamamatsu Photonics’s image viewer software (NDP.view2 Viewing software U12388–01). The density of CALR staining was obtained by evaluating integrated optical density by Color Deconvolution plugin through ImageJ, measured in independent slide images acquired with the same optical microscopic parameters such as magnification, light exposure, and acquisition time. The mean of positive cells detected in 5 fields for each sample was used in the downstream statistical analysis.

For histological characterization, MDOTS were seeded on a layer of Matrigel in 8-well chambers and grown for 5 days at 37 °C and 5% CO_2_. MDOTS were then fixed in 4% PFA at room temperature for 2 hours and subsequently washed with H_2_O. After harvested, MDOTS were transferred into Tissue-Tek®Cryomolds® already coated with histogel base (Epredia HistoGel), and then covered with a further histogel layer. The solidified blocks were transferred into formalin overnight and then in 70% ethanol for 24 hours before embedding in paraffin. Sections of 2 μm were deparaffinized, rehydrated in water and stained with hematoxylin, eosin and synaptophysin.

### Chemokine analysis

Total protein extract (150 μg) quantified by the bicinchoninic acid (BCA) assay (Thermo Fisher Scientific) were used. Chemokines were detected using the Proteome Profiler Mouse Chemokine Array Kit (R&D Systems) according to the manufacturer’s instructions. The signal was detected using Western Lightning ECL Pro (PerkinElmer) and individual chemokine spots quantified using Image Studio Lite software (version 5.2).

### Statistical analysis

Graphpad prism 8.0.2 software was used to calculate significance between the samples. Statistical tests are indicated in each figure legend. Unless specifically stated all data are representative of > 3 separate experiments. Error bars represent SEM and are derived from triplicate experimental conditions. *P* values ≤0.05 were considered significant.

## Results

### Immunomodulatory impact of chemotherapeutic drugs in NB

**Fig. 1:**
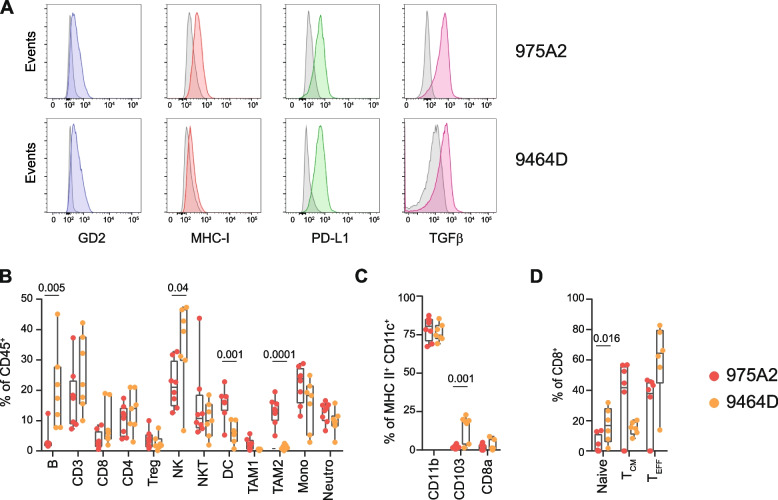
Tumor microenvironment of transplantable NB 975A2 and 9464D mouse models. **A** Representative flow-cytometry histograms of GD2 (blu plot), MHC class I (red plot), PD-L1 (green plot) and TGFβ expression (pink plot) in 975A2 and 9464D tumor cells. Isotype-matched negative control antibody is shown as grey plot. **B**-**D** Flow-cytometry analysis of the immune infiltrate of 50–100 mm^3^-size 975A2 and 9464D tumors grown subcutaneously. Levels of significance for comparison between samples were determined by ANOVA. Statistically significant *P* values are shown

To address the potential impact of chemotherapeutic drugs on immune responses to NB, we chose two syngeneic mouse tumor models, 975A2 and 9464D, derived from spontaneous NB arising in TH-MYCN transgenic mice on C57BL/6 background [27]. We used multi-color flow-cytometry panels and progressive gating strategies to dissect the phenotype of both tumor models and their constitutive tumor-immune infiltrate in tumor masses of 50–100 mm^3^ (Supplementary Fig. S1A and B). Similar to human high-risk NB, both tumor models expressed GD2 tumor antigen [37], low levels of MHC class I [21], and appreciable levels of PD-L1 [21] and TGFβ [25, 38] (Fig. 1A). Tumors were infiltrated by various subsets of lymphoid and myeloid cell populations, with DCs and tumor associated macrophages type 2 (TAM2) significantly more abundant in 975A2, whereas B and NK cells significantly more prevalent in 9464D (Fig. 1B). The other immune cell populations were equally represented in the tumor infiltrates of the two NB models. We characterized the distribution of CD11c^+^ DCs infiltrating tumors by gating on CD45^+^F4/80^−^Ly6G^−^Ly6C^−^MHCII^+^CD11c^+^ with differential expression of CD11b, CD103 and CD8 (Supplementary Fig. S1B). The CD11b^+^ DC subset was the most abundant in both tumor models, whereas CD103^+^ DC subset was predominant in 9464D tumors (Fig. 1C). Next, we addressed to count directly in vivo the percentages of *naïve* (CD44^−^CD62L^+^), central memory (CD44^+^CD62L^+^) and effector memory (CD44^+^CD62L^−^) cells within the pool of CD8^+^ T cells in both tumor models (Supplementary Fig. S1C). N*aïve* and effector memory CD8^+^ T cells were more abundant in 9464D, whereas central memory CD8^+^ T cells were prevalent in 975A2 (Fig. 1D).

**Fig. 2:**
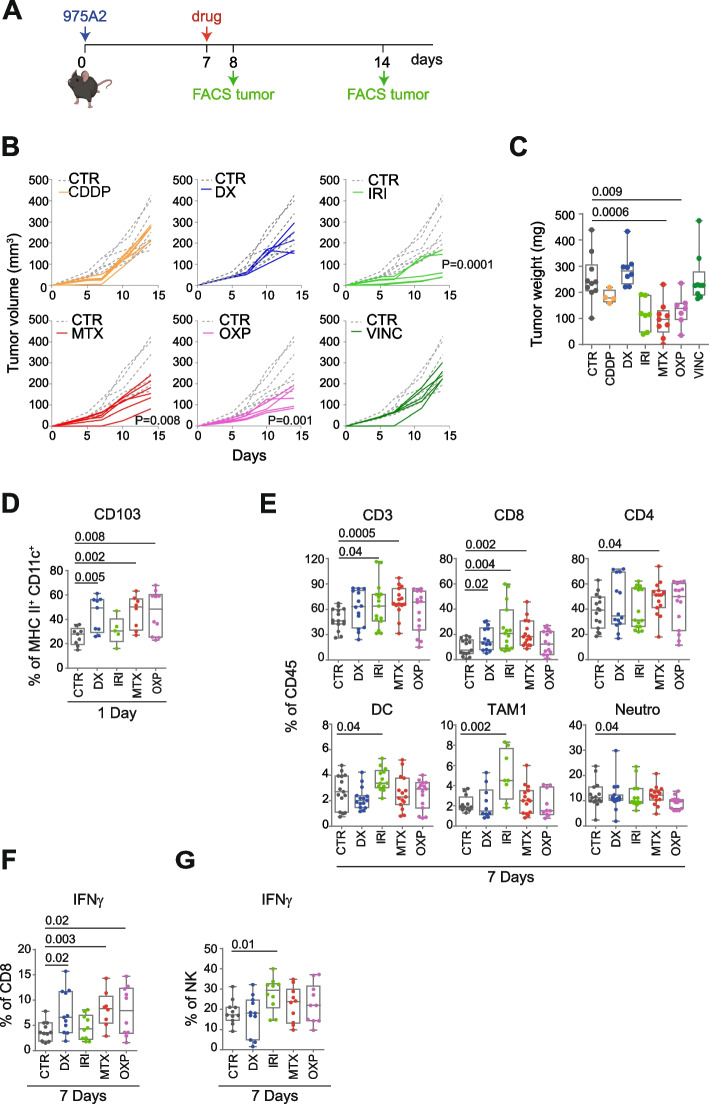
Low-doses chemotherapeutic drugs promote immune cell recruitment and activation of CD8^+^ T cells and NK cells in 975A2 tumors. **A** Schematic representation of the drug treatment and timing of tumor immune infiltrate analysis. **B** Tumor growth of 975A2 injected subcutaneously in C57BL/6 mice treated as indicated. Significance at day 7 after the start of treatment (Mann Whitney test). **C** Weight of explanted tumors at 7 days after the start of treatment. **D**, **E** Flow-cytometry analysis of immune content of 975A2 tumors treated 1 day (**D**) and 7 days (**E**). **F**, **G** Flow-cytometry analysis of IFNγ expression of tumor-infiltrating CD8^+^ T cells (**F**) and NK cells (**G**) in 7 day-treated 975A2 tumors. Levels of significance for comparison between samples were determined by ANOVA. CTR, vehicle control; CDDP, cisplatin; DX, doxorubicin; IRI, irinotecan; MTX, mitoxantrone; OXP, oxaliplatin; VINC, vincristine. Statistically significant *P* values are shown

Collectively, these data indicate that both tumor models are suitable for evaluating the immunomodulatory effect of chemotherapeutic drugs. To this end, we engrafted 975A2 cells in the left flank of syngeneic C57BL/6 mice, and when the tumor size reached 50–100 mm^3^, we treated the mice with a control vehicle or low doses of the selected drugs, including anthracyclines (i.e., DX, MTX and OXP) and those used in the treatment of high-risk NB (i.e., CDDP, IRI and VINC) (Fig. 2A). The 975A2 tumor grew progressively in mice treated with CDDP, DX and VINC, whereas it was significantly attenuated in mice treated with IRI, MTX and OXP (Fig. 2B). Of note, tumors treated with MTX and OXP exhibited significantly reduced tumor weight compared with control tumors 7 days after treatment (Fig. 2C). Mice were sacrificed 1 day or 7 days after treatment and the tumor masses were dissociated and analysed by flow cytometry, distinguishing the immune component (CD45^+^) from the non-immune component (CD45^−^) in terms of the percentage of live cells analysed. Treatment with CDDP and VINC did not alter the immune content of 975A2 tumor (Supplementary Fig. S2), while, on the contrary DX, IRI, MTX and OXP were effective in recruiting different subsets of CD45^+^ immune cells at different times (Fig. 2D and E). Specifically, the CD103^+^ DC subset was significantly increased in tumors treated 24 hours with DX, MTX and OXP, compared to vehicle-treated tumors (Fig. 2D). After 7 days, tumors treated with DX, IRI or MTX were significantly enriched in CD8^+^ T cells (Fig. 2E). Treatment with MTX also induced an increase in CD4^+^ T cells, whereas IRI also caused an increase in DCs and TAM1 (Fig. 2E). OXP treatment was instead associated with a significant reduction of neutrophils (Fig. 2E). Interestingly, treatment with DX, MTX and OXP caused a significant increase in tumor-infiltrating CD8^+^ T cells expressing IFNγ (Fig. 2F). In contrast, IRI treatment caused an increase in IFNγ-producing tumor-infiltrating NK cells (Fig. 2G). None of these treatments affected the other immune cell populations tested (Supplementary Fig. S3).

Collectively, these data indicate that low doses of DX, IRI, MTX, and OXP exert an immunomodulatory effect in the tumor immune microenvironment, facilitating both the recruitment of intratumoral DCs, CD8^+^ T cells and NK cells, and a partial control of tumor growth.

### Low-dose mitoxantrone recalls immune cells by promoting T-cell and NK-cell activation in murine-derived organotypic tumor spheroids when combined with TGFβ and PD-1 blockade

**Fig. 3:**
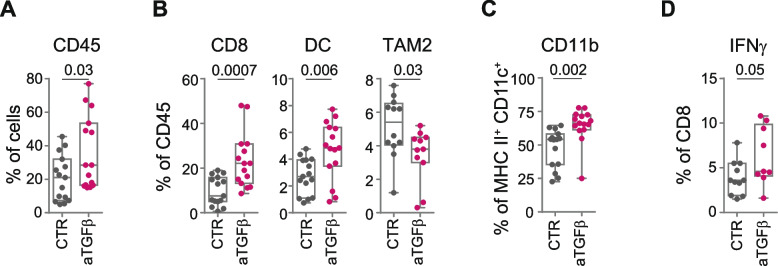
TGFβ blockade recalls activated CD8^+^ T cells in 975A2 tumors. **A-C** Flow-cytometry analysis of immune content in 975A2 tumors 7 days after the start of treatment. **D** Flow-cytometry analysis of IFNγ expression of tumor-infiltrating CD8^+^ T cells in 7 day-treated 975A2 tumors. Levels of significance for comparison between samples were determined by two-tailed Student’s t test. CTR, vehicle control; aTGFβ, anti-TGFβ. Statistically significant *P* values are shown

Recent studies have emphasized the pivotal role of TGFβ as a master regulator of the TME in the development of resistance to ICI [39]. Activation of the TGFβ pathway has been associated with failure to response to ICI in cancer patients resulting from the reduced tumor infiltration of effector CD8^+^ T cells [40, 41]. Indeed, therapeutic co-administration of TGFβ and PD-L1 blockade facilitates T-cell penetration into the tumor center, vigorous antitumor immunity, and tumor regression [42]. Given the key role of TGFβ in TME remodeling in human NB [25, 38, 43], and its expression level in both 975A2 and 9464D tumor models (Fig. 1A), we evaluated the effect of anti-TGFβ in terms of immune cell recall in 975A2-bearing mice (Fig. 3A and Supplementary Fig. S4). Mice with tumor of size 50–100 mm^3^ were intraperitoneally treated with anti-TGFβ or control vehicle and sacrificed 1 day or 7 days after treatment to examine the tumor immune infiltrate. Total immune content (CD45^+^), DC subsets and macrophages were equally represented in tumor infiltrates at 24 hours after treatment (Supplementary Fig. S4A-C). After 7 days, anti-TGFβ treatment was associated with a significant increase in tumor-infiltrating CD45^+^ immune cells, mainly due to CD8^+^ T cells and DCs (i.e., CD11b), and a significant reduction of TAM2 (Fig. 3A-C). Interestingly, TGFβ blockade also induced an increase in CD8^+^ T-cells expressing IFNγ (Fig. 3D). None of the other immune cell populations was affected by TGFβ blockade (Supplementary Fig. S4D-G). These data demonstrate that TGFβ blockade may be an effective strategy to recall CD8^+^ T cells into the TME of NB.

**Fig. 4:**
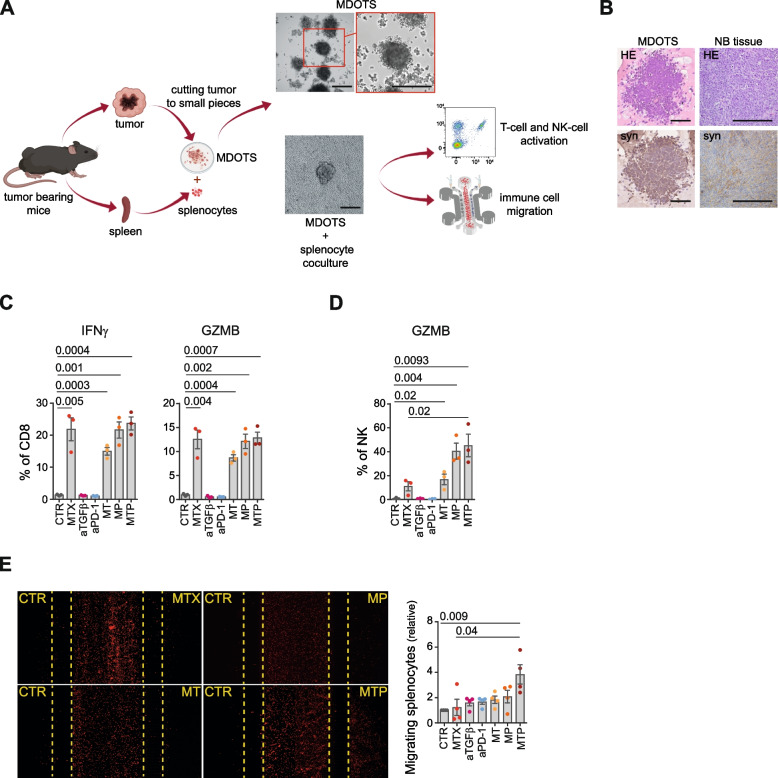
Low-dose mitoxantrone recalls activated CD8^+^ T cells and NK cells in MDOTS when combined with TGFβ and PD-1 blockade. **A** Experimental scheme. Explanted tumors are reduced to small pieces, cultured to form MDOTS and then co-cultured with syngeneic splenocytes from tumor-bearing mice in ULA plates or microfluidic devices. Representative images of MDOTS cultured with or without splenocytes are shown. Original magnification, 20x. Scale bar, 30 μm. **B** Representative IHC staining of hematoxylin and eosin (HE) and synaptophysin (syn) in 975A2 MDOTS and the tumors from which they were derived. Brown, synaptophysin positive cells. Nuclei were counterstained with hematoxylin (blue). Original magnification, 20x. Scale bar, 30 μm. **C**, **D** Flow-cytometry analysis of IFNγ and granzyme B expression of CD8^+^ T cells (**C**) and NK cells (**D**) from splenocytes co-cultured 24 hours with drug-treated and untreated 975A2 MDOTS. **E** Representative images of the migration of red-labeled splenocytes in microfluidic devices to drug-treated and untreated 975A2 MDOTS after 24 hours of co-culture. The number of splenocytes migrating versus drug-treated and untreated 975A2 MDOTS was assessed by ImageJ software. Data are shown as fold change ± SD. Levels of significance for comparison between samples were determined by ANOVA (**C-E**). CTR, vehicle control; MTX, mitoxantrone; aTGFβ, anti-TGFβ; aPD-1, anti-PD-1; MT, mitoxantrone and anti-TGFβ; MP, mitoxantrone and anti-PD-1; MTP, mitoxantrone, anti-TGFβ and anti-PD-1; GZMB, granzyme B. Statistically significant *P* values are shown

Based on these results, we therefore evaluated the possibility of enhancing the immunomodulatory effect of low-doses of DX, IRI, MTX and OXP by simultaneously targeting the immunosuppressive environment of NB by blocking TGFβ and PD-1. To minimize the number of mice required to test multiple combinations, we exploited the ability of explanted NB masses to form MDOTS, three-dimensional cellular structures which recapitulates the features of the original tumor and its tumor microenvironment (Fig. 4A) [28]. Indeed, IHC analysis revealed significant similarity between MDOTS and murine tissues of origin in terms of both morphology, cytoarchitecture and expression of synaptophysin, a specific marker for NB diagnosis (Fig. 4B). 975A2 MDOTS were treated with the selected immunomodulatory drugs in combination with anti-TGFβ and/or anti-PD-1 antibodies, and then co-cultured with splenocytes from tumor-bearing mice, both in ULA plates and microfluidic devices to study the effect on the functional status of CD8^+^ T cells and NK cells and the dynamic interactions between immune and tumor cells [44], respectively (Fig. 4 and Supplementary Fig. S5). MDOTS treated with MTX either alone or in combination with anti-TGFβ antibody (abbreviated to MT), anti-PD-1 antibody (abbreviated to MP) or both anti-TGFβ and anti-PD-1 antibodies (abbreviated to MTP) were able to stimulate the production of IFNγ and granzyme B by CD8^+^ T cells (Fig. 4C). Interestingly, MTX in combination with TGFβ and/or PD-1 blockades was also able to stimulate granzyme B production by NK cells (Fig. 4D). In contrast, none of the other drugs tested (DX, IRI and OXP) was able to induce an increase in CD8^+^ T-cell or NK-cell activation status (Supplementary Fig. S5A-C).

To evaluate the effect of drugs on immune cell recall, red-labeled splenocytes were loaded into the central chamber of the microfluidic devices (Supplementary Fig. S5D). Drug-treated and untreated MDOTS were loaded into the side chambers of the device (Supplementary Fig. S5D). Treatment of MDOTS with MTP induced a significant recruitment of immune cells compared to control and MTX-treated MDOTS (Fig. 4E). Increased recruitment of immune cells was also obtained with DX in combination with TGFβ and PD-1 blockades, and OXP in combination with TGFβ (Supplementary Fig. S5E-G).

Collectively, these data indicate that i) treatment of MDOTS with immunomodulatory drugs in combination with TGFβ and PD-1 blockades induces a significant increase in immune cell recruitment, ii) MTX alone stimulates IFNγ and granzyme B production by CD8^+^ T cells, and iii) MTX in combination with TGFβ and/or PD-1 blockades stimulated NK cells to produce granzyme B.

### Low-dose mitoxantrone delays the growth of transplanted tumors and reshapes the intratumoral infiltrate when combined with TGFβ and PD-1 blockades

**Fig. 5:**
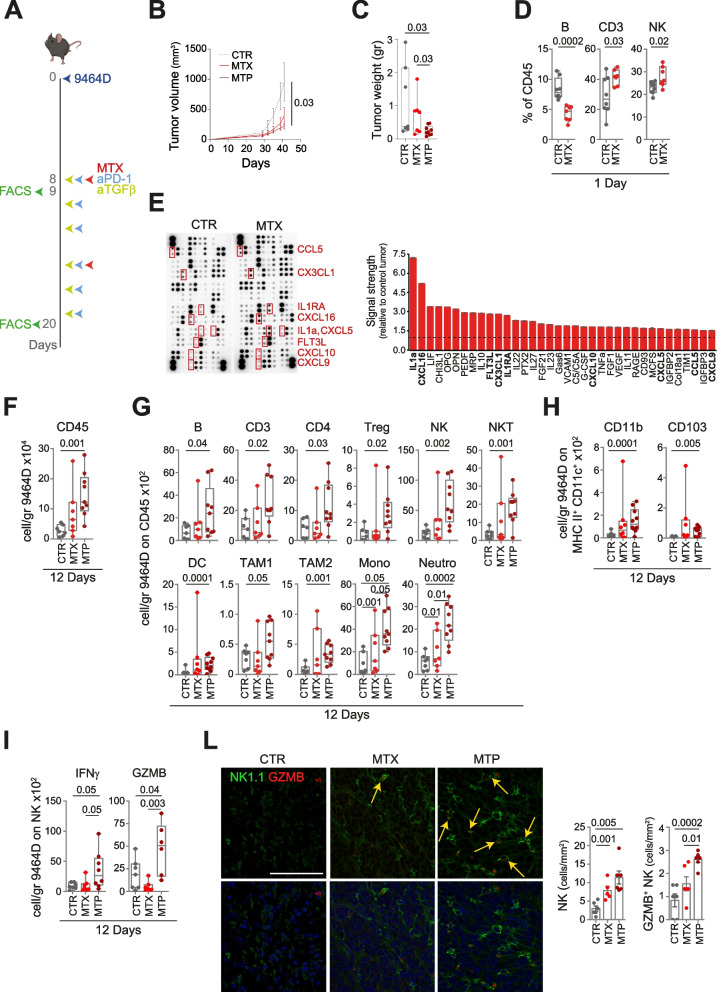
Treatment of low-dose mitoxantrone in combination with TGFβ and PD-1 blockade delays the growth of subcutaneously transplanted 9464D tumors and reshapes the intratumoral infiltrate. **A** Schematic representation of the drug treatment and timing of tumor immune infiltrate analysis. **B** Tumor growth of 9464D injected subcutaneously in C57BL/6 mice and treated as indicated. Significance at day 41 (Mann Whitney test). **C** Weight of explanted tumors at day 41 after cell inoculation. **D** Flow-cytometry analysis of the immune compartment in 1 day-MTX-treated 9464D tumors. Levels of significance for comparison between samples were determined by two-tailed Student’s t test. **E** Chemokine expression in 1 day-MTX-treated 9464D lysates by protein array. Relative chemokine expression based on densitometric analysis is shown on the right. **F-H** Flow-cytometry analysis of the immune content in 9464D tumors treated for 12 days. **I** Flow-cytometry analysis of the activation status of tumor-infiltrating NK cells. **L** Multiple immunofluorescence staining of drug-treated 9464D tumor specimens for NK1.1 (green) and granzyme B (red), shown at original magnification × 40 (zoom), scale bar 30 μm. Images with nuclei (Hoechst) are shown in the bottom panel. Granzyme B-positive NK cells are indicated by yellow arrows. Quantitative analysis of the indicated immune cells from *n* = 6 biologically independent 9464D specimens is shown on the right. Levels of significance for comparison between samples in **F-L** were determined by ANOVA. CTR, vehicle control; MTX, mitoxantrone; MTP, mitoxantrone, anti-TGFβ and anti-PD-1; GZMB, granzyme B. Statistically significant, *P* values are shown

To define the in vivo antitumor effect of MTP, we engrafted both 9464D and 975A2 models into the left flank of syngeneic C57BL/6 mice. Administration of low-dose MTX was initiated in mice with tumor sizes 50–100 mm^3^ and was repeated after 1 week. Anti-TGFβ and/or anti-PD-1 antibodies were adimistrated according to the scheme shown (Fig. 5A and Supplementary Fig. S6A). Treatment with 2 cycles of MTX alone caused a slowing of growth of both tumors (Fig. 5B and Supplementary Fig. S6B). When MTX was administered in combination with anti-TGFβ (MT) or anti-PD-1 (MP), the control of tumor growth was not improved (Supplementary Fig. S7A and B). Interestingly, MTP treatment conferred complete control of tumor growth in all mice, with a significant reduction in both tumor volume and weight compared to control and MTX-treated mice (Fig. 5B, C and Supplementary Figs. S6B, C and S7A, B).

We then compared the immune infiltrate in tumors treated with low-dose MTX alone or with MTP. At the early time point (24 hours after the first administration), MTX treatment was associated with a significant increased expression of calreticulin, an important hallmark of ICD (Supplementary Fig. S8) [45]. Treatment with MTX induced a significant reduction of B cells in both tumor models (Fig. 5D and Supplementary Fig. S6D). MTX-treated 9464D tumors were also significantly enriched in CD3^+^ T cells and NK cells (Fig. 5D), whereas MTX-treated 975A2 contained an increased number of NKT cells and CD103^+^ and CD8a^+^ DC subsets and reduced CD11b^+^ DC subset (Supplementary Fig. S6D, E). The mouse cytokine array was used to determine whether MTX treatment was able to stimulate the release of chemokines involved in immune cell trafficking. Lysates of MTX-treated 9464D tumors displayed a more than 2.5-fold increase of IL1a, CXCL16, FLT3L, CX3CL1 and IL1RA, and a more than 1.5-fold increase of CXCL10, CXCL5, CCL5 and CXCL9 compared to control tumor lysates (Fig. 5E). Similarly, lysate of MTX-treated 975A2 tumors displayed a more than 1.5 fold increase of CCL5, FLT3L, CD160 and CXCL5 (Supplementary Fig. S6F). At a later time point (12 days from the first drug administration), MTX alone induced a significant increase of monocytes and neutrophils in both tumor models (Fig. 5G and Supplementary Fig. S6H). 975A2 tumors treated with MTX also showed an enrichment of CD45^+^ cells, B cells, TAM2 and CD8^+^ T cells, as well as CD8^+^ T cells expressing IFNγ and NK cells expressing IFNγ and granzyme B (Supplementary Fig. S6G, H, L and M). Administration of MTP had an even stronger impact compared to MTX alone on both the lymphoid and myeloid compartments (Fig. 5F-I and Supplementary Fig. S6G-M). Specifically, unlike MTX alone, combination treatment induced a highly significant increase of CD45^+^ cells, NK cells, DCs, TAM1, monocytes, neutrophils and CD11b^+^ and CD103^+^ DC subsets compared to control mice in both tumor models (Fig. 5F-H and Supplementary Fig. S6G-I). MTP also induces a significantly enrichment in B cells, T cells (CD3, CD4 and Treg) and NKT cells in 9464D (Fig. 5G), and TAM2 and IFNγ-expressing CD8^+^ T cells in 975A2 tumors (Supplementary Fig. S6H and L). Interestingly, MTP made both tumors more infiltrated by IFNγ- and granzyme B-expressing NK cells, and in 9464D the increase was statistically significant not only compared with control, but also compared with MTX alone (Fig. 5I and Supplementary Fig. S6M). None of these drug treatments affected the other immune cell populations tested (Supplementary Fig. S9).

**Fig. 6:**
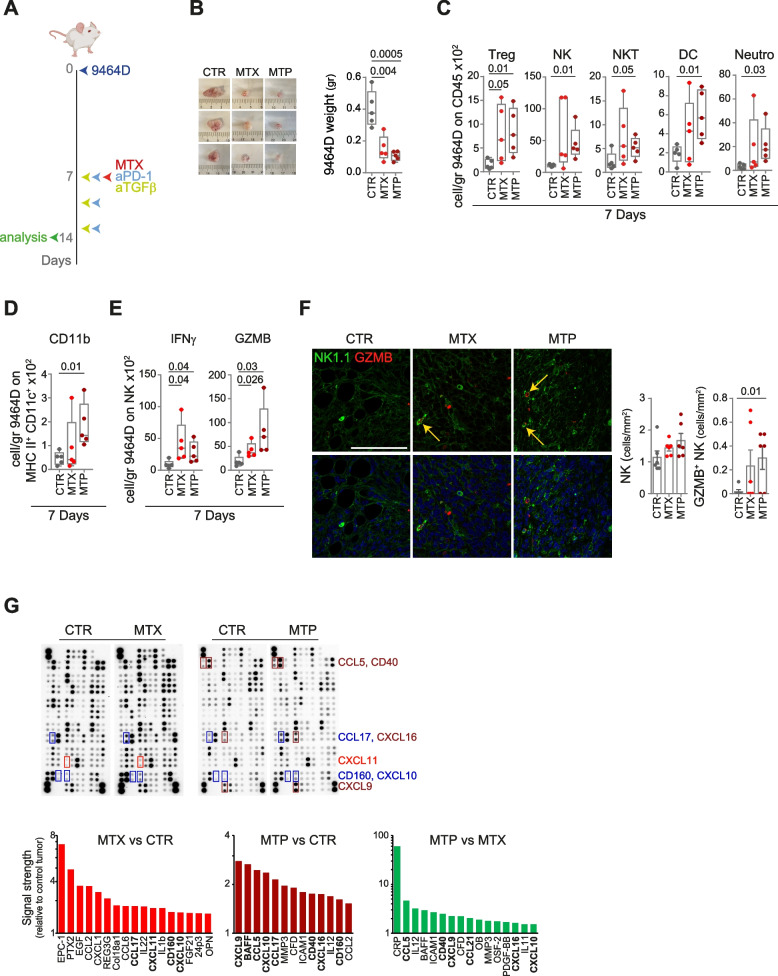
Treatment with low-dose mitoxantrone in combination with TGFβ and PD-1 blockade delays the growth of orthotopically transplanted tumors and reshapes the intratumoral infiltrate. **A** Schematic representation of the drug treatment and timing of tumor immune infiltrate analysis. **B** Representative images and weight of explanted tumors at day 7 after the start of treatment. **C**-**E** Flow-cytometry analysis of the immune content (**C**, **D**) and activation status of tumor-infiltrating NK cells (**E**) in 7 day-treated 9464D tumors. **F** Multiple immunofluorescence staining of drug-treated 9464D tumor specimens for NK1.1 (green) and granzyme B (red) shown at original magnification × 40 (zoom), scale bar 30 μm. Images with nuclei (Hoechst) are shown in the bottom panel. Granzyme B-positive NK cells are indicated by yellow arrows. Quantitative analysis of the indicated immune cells from n = 6 biologically independent 9464D specimens is shown on the right. Levels of significance for comparison between samples in **C-F** were determined by ANOVA. **G** Chemokine expression in 7 day-drug-treated 9464D lysates by protein array. Relative chemokine expression based on densitometric analysis is shown on the right. CTR, vehicle control; MTX, mitoxantrone; MTP, mitoxantrone, anti-TGFβ and anti-PD-1; GZMB, granzyme B. Statistically significant *P* values are shown

Next, we evaluated the effectiveness of the combined treatment also in luc-expressing 9464D cells engrafted into the adrenal gland of C57BL/6 albino mice. Treatment was initiated in mice with large established tumors (BLI > 1 × 10^7^) following the schedule shown in Fig. 6A. Mice were sacrificed seven days after the first administration to assess immune content and chemokine expression. MTX treatment resulted in a significant reduction in tumor weight compared to control mice which was further enhanced by MTP treatment (Fig. 6B). Analysis of the immune content revealed a significant increase in the number of Tregs and NK cells expressing IFNγ and granzyme B in tumors treated with MTX alone (Fig. 6C-E). Similar to subcutaneous tumors (Fig. 5G), administration of MTP resulted in a significant increase in Treg, NK, NKT, DC (i.e., CD11b), and neutrophils (Fig. 6C and D). MTP-treated 9464D tumors were also significantly enriched in NK expressing IFNγ and granzyme B (Fig. 6E). Interestingly, IF analyses of MTP-treated tumors confirmed a significant increase of tumor-infiltrating NK cells expressing granzyme B (Fig. 6F). None of the other immune cell populations tested was affected by treatments with MTX alone or in combination (Supplementary Fig. S10). A protein array on a lysate of tumors treated with MTX alone displayed a more than 1.5 fold increase of CCL17, CXCL11, CD160 and CXCL10, as compared to control tumor lysate (Fig. 6G). Lysate from MTP-treated tumors showed an even stronger upregulation of chemokines involved in immune cell recruitment, including CXCL9, CCL5, CXCL10, CD40 and CXCL16, compared to both control and MTX-treated tumor lysates (Fig. 6G).

Collectively, these data show that treatment with low-dose MTX in combination with TGFβ and PD-1 blockade drives the remodeling of myeloid and lymphoid compartments supported by inflammatory cytokine production. Notably, MTP treatment results in a significant increase in intratumoral DC and NK cells expressing granzyme B, and lead to the control of tumor growth in these aggressive NB mouse models.

### Low-dose MTP induces immune cell recruitment into PDOTS and increased granzyme B production by CD8^+^ T cells and NK cells

**Fig. 7:**
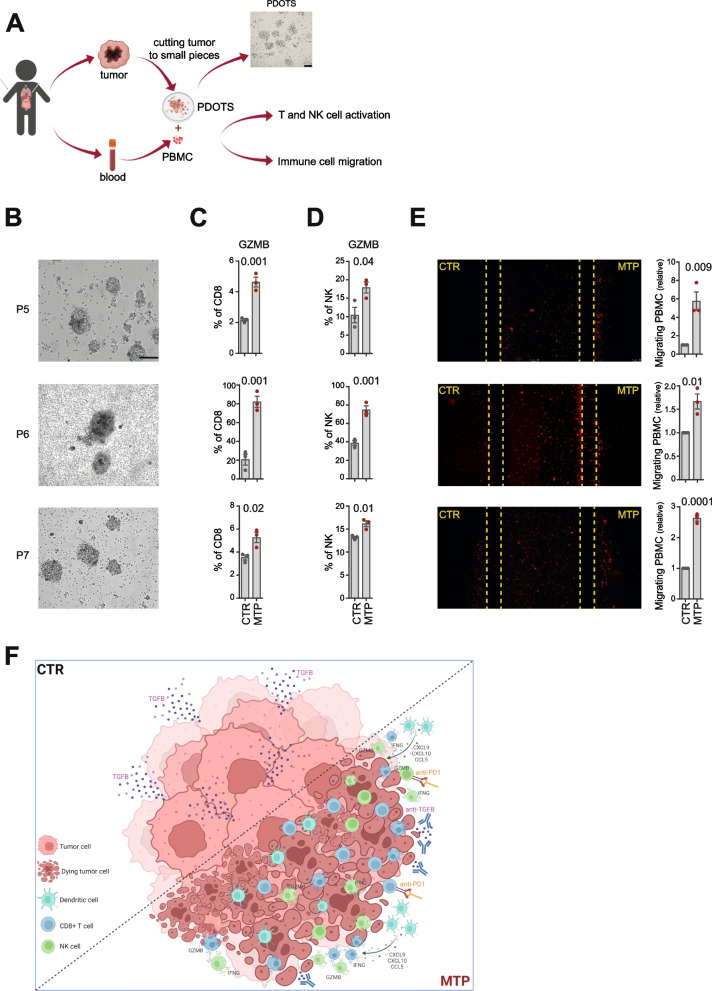
Low-dose mitoxantrone recalls activated CD8^+^ T cells and NK cells in PDOTS when combined with TGFβ and PD-1 blockade. **A** Experimental scheme. Explanted human tumors are reduced to small pieces, cultured to form PDOTS and then co-cultured with autologous PBMC in ULA plates or microfluidic devices. A representative image of PDOTS cultured in ULA plate is shown. Original magnification, 20x. Scale bar, 30 μm. **B** Representative images of PDOTS derived from P5, P6 and P7 NB patients, respectively. Original magnification, 20x. Scale bar, 30 μm. **C**, **D** Flow-cytometry analyses of granzyme B expression by CD8^+^ T cells (**C**) and NK cells (**D**) from autologous PBMC co-cultured 24 hours with drug-treated PDOTS. **E** Representative images of the migration of red-labeled autologous PBMCs in microfluidic devices to drug-treated and untreated PDOTS after 24 hours of co-culture. The number of PBMCs migrating versus drug-treated and untreated PDOTS was assessed by ImageJ software. Data are shown as fold change ± SD. Levels of significance for comparison between samples were determined by two-tailed Student’s t test. **F** A schematic representation depicting the transition from an immunosuppressive (CTR) to a tumor friendly microenvironment driven by MTP treatment. CTR, vehicle control; MTX, mitoxantrone; MTP, mitoxantrone, anti-TGFβ and anti-PD-1; GZMB, granzyme B. Statistically significant *P* values are shown

Finally, we evaluated the immunomodulatory effect of low-dose MTP in human NBs. PDOTS successfully generated from surgically removed NB tissues were treated with MTP or untreated, and then co-cultured with autologous PBMCs in ULA plates or microfluidic devices (Fig. 7A). Interestingly, MTP treatment induced a significant increase in granzyme B production by both CD8^+^ T cells and NK cells, as well as a significant recruitment of immune cell in 3 of the 4 PDOTS analysed (Fig. 7B-E and Supplementary Fig. S11).

In conclusion, these data provide proof of principle that the proposed combinatorial strategy may serve as a potential immunomodulatory therapy for NB.

## Discussion

This study demonstrates that low-dose MTX curbs in vivo NB growth leading to substantial tumor regression and remodeling of the tumor’s immune landscape when combined with PD-1 and TGFβ blockade. This combined treatment is also able of inducing the recruitment of immune cells and the activation of CD8^+^ T cells and NK cells against PDOTS generated from human NB patients.

ICIs have been extensively shown to trigger T-cell effector function to control tumor growth in both mice and human cancers [46, 47]. However, antitumor immune responses to individual immunotherapeutic agents remain limited to a subset of patients [1]. This is because immunotherapy is ineffective for tumors that, as high-risk NB, are both lacking immune cells and characterized by an immunosuppressive TME [1]. Therefore, developing combination therapies to induce recruitment of immune cells and target the immunosuppressive factors released in the TME could be a successful strategy to improve the efficacy of treatment regimens [48].

Chemotherapy agents, originally known to directly inhibit or kill malignant cells, have recently been found to promote antitumor immunity by increasing tumor immunogenicity, enhancing T-cell infiltration, or reducing immunosuppressive cell populations [49]. Some of these chemotherapy drugs are known to activate tumor-specific T cells by inducing ICD when administered at low doses [7]. The effectiveness of these drugs increases in association with immune checkpoint therapies [50]. Indeed, combined treatment of DX with PD-1 or PD-L1 antibodies resulted in a significant increase in efficacy in metastatic triple-negative human breast cancer and in various murine tumors, such as melanoma and breast cancer [51, 52]. Similarly, OXP treatment has been found to increase the efficacy of anti-PD-L1 therapy in murine colorectal cancer [53].

NB cells are capable of secreting a variety of soluble mediators that can suppress lymphocyte activation, including TGFβ [23, 38, 54]. Indeed, high levels of tumor TGFβ have been associated with reduced event free survival [43]. Activation of the TGFβ pathway has also recently emerged as a potential factor responsible for primary resistance to immune checkpoint blockade therapy [11, 12]. Combined anti-TGFβ/anti-PD-1 treatment led to profound and durable antitumor responses in urothelial, melanoma, and breast cancer models, and promoted the establishment of immunological memory in a tumor rechallenge model [55, 56]. The induction of long-term tumor-specific T-cell memory is attributed to immune checkpoint PD-1 and PD-Ll blockade [55, 56]. In this context, TGFβ blockade has been found to restore an immunity-friendly environment in NB capable of unleashing the full potential of reactive immune cells and increasing their persistence [57]. Based on these considerations, here we evaluated the immunomodulatory effect of 6 chemotherapy agents administered at low doses, including those used in the therapy of high-risk NB and/or able to induce ICD, in two transplantable 9464D and 975A2 murine models that closely recapitulate the molecular and biological features of high-risk human NBs [27, 58–61]. Low-dose MTX was found to have the greatest immunostimulatory capacity able to attract immune cells into the NB microenvironment in ex-vivo and in vivo approaches. To date, MTX has been approved for the treatment of several tumors, including acute non-lymphoblastic leukemia and some advanced forms of prostate cancer (clinicaltrials.gov). We found that low-dose MTX triggers T-cell priming in NB through induction of ICD [62], making the tumor more susceptible to immune-mediated attack by up-regulating the expression of MHC class I molecules (Supplementary Fig. S12). Interestingly, low-dose MTX also results in a concomitant increase in PD-L1 levels (Supplementary Fig. S12), thus strengthening the idea that chemotherapy alone may not provide a lasting therapeutic effect. This finding is consistent with the recent evidence indicating that treatment regimens combining multiple immunotherapeutic strategies, some of which even engaging innate and adaptive immunity, are more effective in NB patients than monotherapies [63–65]. We found that low-dose MTX in combination with TGFβ and PD-1 blockers is able to remodeling the landscape of tumor-infiltrating immune cells by compensating for the lack of immune cells that characterize high-risk NBs [20–22] (Fig. 7F). Consistently, dual PD-1/TGFβ blockade has recently been shown to i) make human tumor cells more sensitive to different chemotherapeutic agents by altering their plasticity [66]; ii) enhance the cytolytic activity of NK and T cells towards tumor cells [67–69]; and iii) up-regulate the expression of immune response genes, including those encoding multiple chemokines, such as CCL5, associated with immune cell infiltration and enhanced anti-tumor activity [70]. Indeed, MTP treatment resulted in an enrichment of DCs and activated CD8^+^ T cells and NK cells in both mouse models of NB, supporting the key role of these immune cells in controlling NB growth, as observed in human NB speciments [20, 22]. We noted that this treatment also induced an enrichment of immunosuppressive populations, such as Treg and neutrophils. The increase of Tregs is unexpected considering the role of TGFβ in maintaining peripheral Treg cells [71]. We interpret this increase as indicative of a strong immune response that results in the recruitment of immune cells, including Treg, to sites of inflammation. As seen for other tumors, the presence of activated effector cells is indicative that the increase in Treg is not sufficient to create a substantially immunosuppressive TME [39]. Like macrophages, murine neutrophils are distinguished into antitumor and protumor neutrophils [72–74]. In the early phase of tumorigenesis, neutrophils appear to contribute to the antitumor immune response, perhaps through stimulation of adaptive immunity and activation of CD8^+^ T cells [72]. Cross-talk between neutrophils and activated T cells resulted in substantial upregulation of the costimulatory molecules on the surface of neutrophils, capable of enhancing T-cell proliferation in a positive feedback loop [75].

Recent evidence highlighted that, even if the adaptive immune system is compromised [76], or T-cell function cannot be fully recovered by PD-1 inhibitors under specific circumstances [77], PD-1/PD-L1 inhibitors can still increase antitumor efficacy. This is because other types of immune cells, such as DCs, TAMs and NK cells, are also responsive to PD-1/PD-L1 antagonists [76, 78–82], thus strengthening the use of these agents to increase antitumor efficacy.

In this regard, we can assume that the addition of anti-TGFβ along with checkpoint blockade interrupts a hierarchy of immunosuppressive events, consisting of TGFβ that dampens the initial immune response by preventing immune cells from infiltrating tumors, and PD-1/PD-L1 signaling that operating at a later stage suppresses the effector functions of immune cells causing their depletion [83].

Immune checkpoint blockade is known to reactivate pre-existing intratumoral T cells in human cancer lesions. This reactivation is accompanied by an increase in the chemoattractant production by different immune cell populations [4, 46, 84]. Interestingly, we noted that, compared with MTX alone, treatment with MTP resulted in a simultaneously increase of different chemokines involved in the recruitment of both lymphoid and myeloid cell populations (Fig. 6G). In addition to promoting recruitment and effector function of tumor-specific CD8^+^ T cells, these chemokines are also able to enrich the TME of functional DC and NK cells, two key immune components associated with improved survival of both adult and pediatric cancer patients [5]. Particularly important is the increase of CCL5, produced by CD8^+^ T cells, NK cells and innate lymphoid cells, that is crucial for recruitment of cDC1s, macrophages and Treg cells in the TME [85]; CXCL9, CXCL10 and CXCL16, produced by DCs and macrophages, that induce the recruitment and activation of NK cells, NKT cells and CD8^+^ T cells [5, 86–88]; CCL21, that significantly increase the proportion of T cells, NK cells and DCs within the tumor [89]; CD40 and FLT3L, produced by intratumoral NK cells, which supports the viability and functions of cDC1s within the TME by promoting their local differentiation from precursor cells [19, 90]. Interestingly, CXCL9 expression by cDCs has been previously described to mediate the clustering of DC-T cells within lymph nodes [91], and since interaction between these two immune cell populations is quite rare in tumors [22, 92], it is possible that increased CXCL9 expression by facilitating these interactions may promote T-cell effector function.

Finally, our findings in human NB specimens strengthen the evidence found in mouse models, providing proof of principle that the proposed combinatorial strategy may serve as a potential alternative therapy in the NB clinic. So far, the treatment protocols under investigation have mostly been derived from therapeutic regimens formulated for adult tumors [93]. This is a major limitation because childhood tumors are genetically different from their adult counterparts [94], suggesting the need for alternative therapeutic approaches. Up to now, few studies have combined immunotherapy and chemotherapy in pediatric cancers, mainly due to the original idea that chemotherapy being immunosuppressive may act by inhibiting the beneficts achieved with immunotherapy [95]. Instead, we believe that the use of metronomic low dose of chemotherapeutics such as MTX, capable of exposing the host immune system to large amounts of tumor antigens and damage-associate molecular patterns (DAMPs), could overcome the relative coldness of childhood cancer, as high-risk NB. Therefore, this study provides a rational approach based on intra-tumoral recall of immune effector cells that allows for greater efficacy of the proposed immunotherapy [96, 97]. Further investigation in a prospective study with a larger number of human NB samples, will be crucial to confirm therapeutic efficacy of MTP treatment.

## Conclusion

The results of this study show that low-dose MTX in combination with TGFβ and PD-1 blockade remodels intratumoral immune cell type and density and control tumor growth, thus suggesting its use in patients with poorly infiltrated T-cell tumors refractory to immunotherapy, such as high-risk NBs.

## Supplementary Information

**Additional file 1: Fig. S1.** Flow-cytometry analysis of tumor-infiltrating immune cells in mouse NB models. **Fig. S2.** Immune content of cisplatin- and vincristine-treated 975A2 tumors. **Fig. S3.** Immune content of drug-treated 975A2 tumors. **Fig. S4.** Effect of anti-TGFβ treatment on immune content of 975A2 tumors. **Fig. S5.** Effect of combined drug treatment on the recall and activation of CD8+ T cells and NK cells. **Fig. S6.** Treatment of mitoxantrone in combination with TGFβ and PD-1 blockade delays growth of subcutaneously transplanted 975A2 tumors and reshapes intratumoral immune infiltrate. **Fig. S7.** Tumor growth of drug-treated 975A2 and 9464D. **Fig. S8.** Expression of calreticulin in 9464D tumors. **Fig. S9.** Immune content of drug-treated 9464D and 975A2 tumors. **Fig. S10.** Immune content of drug-treated 9464D grown in the adrenal gland. **Fig. S11.** Mitoxantrone recalls activated CD8+ T cells and NK cells in some PDOTS when combined with TGFβ and PD-1 blockade. **Fig. S12.** Mitoxantrone TME of drug-treated 9464D grown in the adrenal gland. **Table S1.** List of antibodies. **Table S2.** Clinical and genetic characteristics of 7 newly diagnosed NB patients.

## Data Availability

The authors declare that all data supporting the findings of this study are available in the main text and supplementary materials. Any other relevant data and code are available from the corresponding author upon reasonable request.

## Abbreviations

ICI: Immune checkpoint inhibitors
DC1: Conventional type 1 dendritic cells
NK: Natural killer cells
TME: Tumor microenvironment
ICD: Immunogenic cell death
NB: Neuroblastoma
IHC: Immunohistochemistry
DX: Doxorubicin hydrochloride
MTX: Mitoxantrone dihydrochloride
OXP: Oxaliplatin
CDDP: Cisplatin
VINC: Vincristine
IRI: Irinotecan
MDOTS: Murine-derived organotypic tumor spheroids
ULA: Ultra-low attachment
PDOTS: Patient-derived organotypic tumor spheroids
INRG: International Neuroblastoma Risk Group Staging System
INPC: International Neuroblastoma Pathology Classification
PBMC: Peripheral blood mononuclear cells
PDMS: Polydimethylsiloxane
IF: Immunofluorescence
DAMPs: Damage-associated molecular patterns
IFN: Type I interferon
PRRs: Pattern recognition receptors
BCA: Bicinchoninic acid
TAM: Tumor associated macrophages
MTP: MTX /anti-TGFβ/anti-PD-1
MT: MTX/anti-TGFβ
MP: MTX/anti-PD-1;
CTR: Control
HE: Hematoxylin and eosin
syn: Synaptophysin
GZMB: Granzyme B
