# Supplementary material for: Combined mitoxantrone and anti-TGFβ treatment with PD-1 blockade enhances antitumor immunity by remodelling the tumor immune landscape in neuroblastoma
Source: J Exp Clin Cancer Res. 2022 Nov 17;41:326. doi: 10.1186/s13046-022-02525-9 (PMC9670422; doi:10.1186/s13046-022-02525-9)
Supplement: Supplementary file 1 — Additional file 1: Fig. S1. Flow-cytometry analysis of tumor-infiltrating immune cells in mouse NB models. Fig. S2. Immune content of cisplatin- and vincristine-treated 975A2 tumors. Fig. S3. Immune content of drug-treated 975A2 tumors. Fig. S4. Effect of anti-TGFβ treatment on immune content of 975A2 tumors. Fig. S5. Effect of combined drug treatment on the recall and activation of CD8+ T cells and NK cells. Fig. S6. Treatment of mitoxantrone in combination with TGFβ and PD-1 blockade delays growth of subcutaneously transplanted 975A2 tumors and reshapes intratumoral immune infiltrate. Fig. S7. Tumor growth of drug-treated 975A2 and 9464D. Fig. S8. Expression of calreticulin in 9464D tumors. Fig. S9. Immune content of drug-treated 9464D and 975A2 tumors. Fig. S10. Immune content of drug-treated 9464D grown in the adrenal gland. Fig. S11. Mitoxantrone recalls activated CD8+ T cells and NK cells in some PDOTS when combined with TGFβ and PD-1 blockade. Fig. S12. Mitoxantrone TME of drug-treated 9464D grown in the adrenal gland. Table S1. List of antibodies. Table S2. Clinical and genetic characteristics of 7 newly diagnosed NB patients. [file 13046_2022_2525_MOESM1_ESM.pdf]

Figure S1

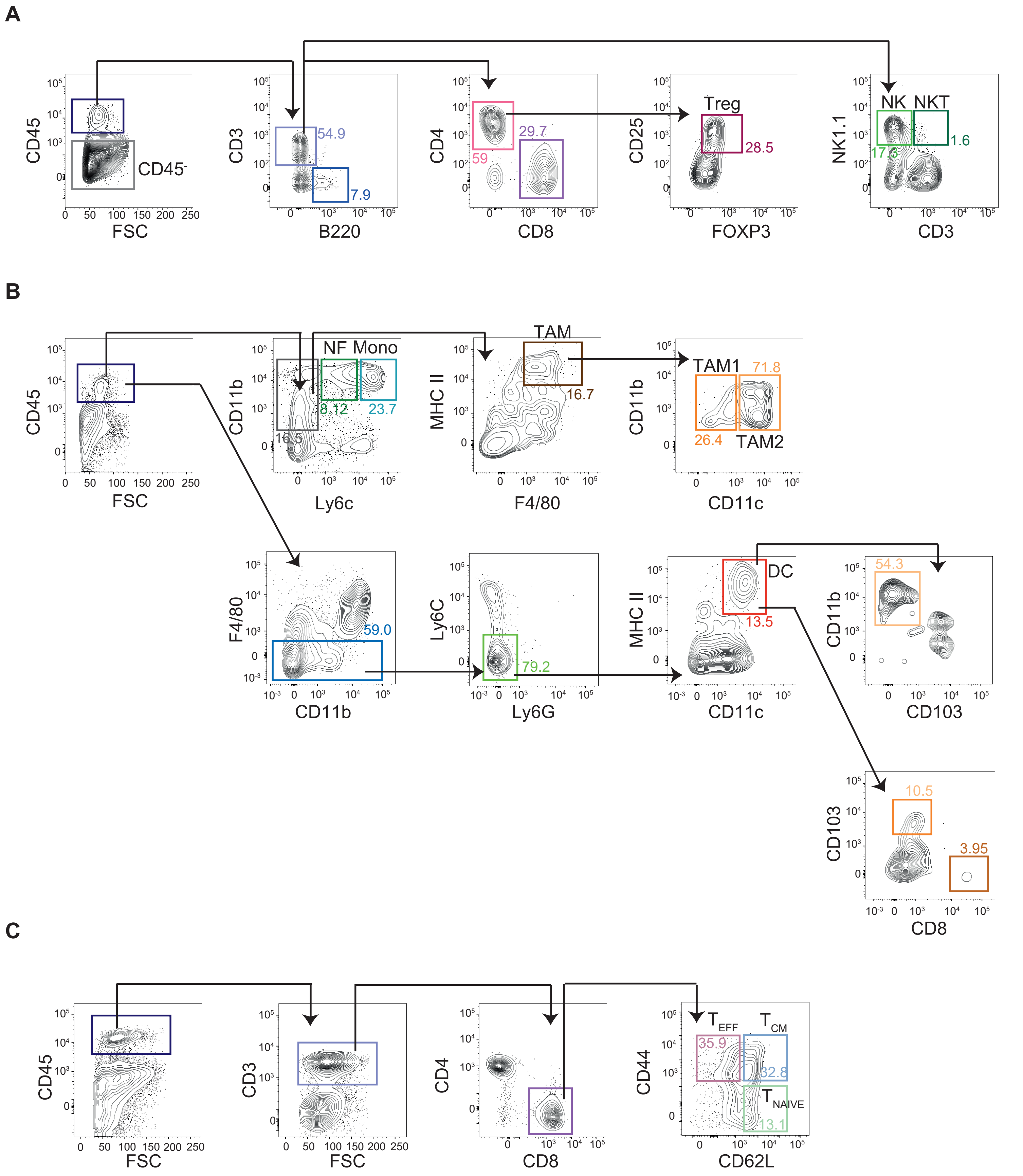
A
CD45
FSC
CD45-
CD3
B220
54.9
7.9
CD4
CD8
59
29.7
CD25
FOXP3
Treg
28.5
NK1.1
CD3
NK
NKT
17.3
1.6
B
CD45
FSC
CD11b
Ly6c
NF
Mono
16.5
8.12
23.7
MHC II
F4/80
TAM
16.7
CD11b
CD11c
TAM1
TAM2
71.8
26.4
F4/80
CD11b
59.0
Ly6C
Ly6G
79.2
MHC II
CD11c
DC
13.5
CD11b
CD103
54.3
CD103
CD8
10.5
3.95
C
CD45
FSC
CD3
FSC
CD4
CD8
CD44
CD62L
T EFF
T CM
T NAIVE
35.9
32.8
13.1

Figure S1, related to Figure 1. Flow-cytometry analysis of tumor-infiltrating immune cells in mouse NB models. Representative flow cytometric gating strategy for 975A2 tumors to define tumor-infiltrating lymphoid cells (**A**), myeloid cells (**B**) and naïve and memory T cells (**C**).

Figure S2

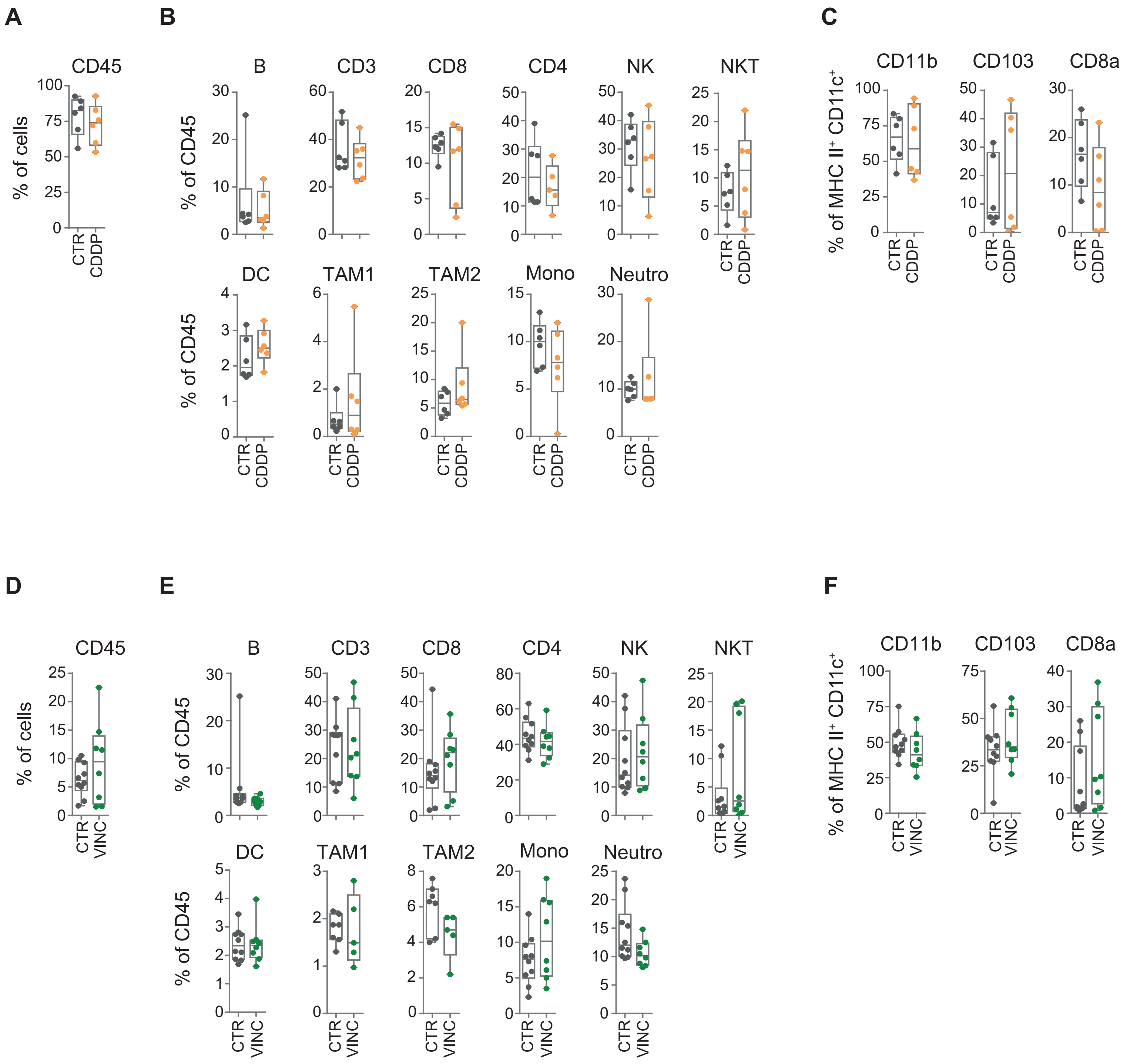

A
B
C
CD45
% of cells
CTR
CDDP
B
CD3
CD8
CD4
NK
NKT
% of CD45
CD11b
CD103
CD8a
% of MHC II+ CD11c+
DC
TAM1
TAM2
Mono
Neutro
D
E
F
VINC

Figure S2, related to Figure 2. Immune content of cisplatin- and vincristine-treated 975A2 tumors. Flow-cytometry analysis of the immune compartment in 975A2 tumors treated with cisplatin (**A-C**) and vincristine (**D-F**) for 7 days. Levels of significance for comparison between samples were determined by two-tailed Student's t test. CTR, vehicle control; CDDP, cisplatin; VINC, vincristine. Non-significant data are shown.

Figure S3

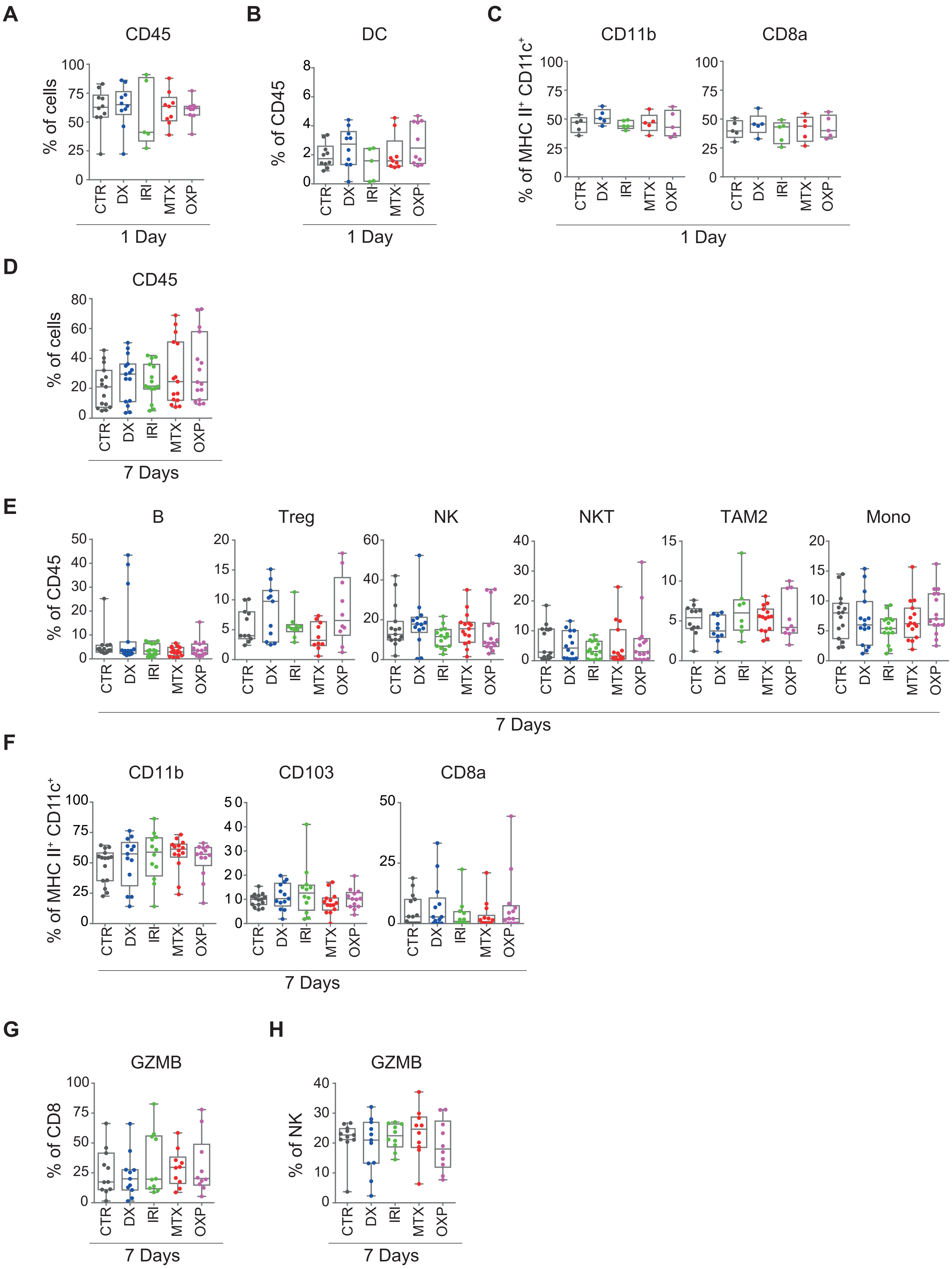
A
CD45
% of cells
CTR
DX
IRI
MTX
OXP
1 Day
B
DC
% of CD45
1 Day
C
CD11b
CD8a
% of MHC II+ CD11c+
1 Day
D
CD45
% of cells
7 Days
E
B
Treg
NK
NKT
TAM2
Mono
% of CD45
7 Days
F
CD11b
CD103
CD8a
% of MHC II+ CD11c+
7 Days
G
GZMB
% of CD8
7 Days
H
GZMB
% of NK
7 Days

Figure S3 related to Figure 2. Immune content of drug-treated 975A2 tumors. Flow-cytometry analysis of the immune content in 975A2 tumors 1 day (**A-C**) and 7 days (**D-F**) after treatment with the indicated drugs. (**G-H**) Flow-cytometry analysis of the granzyme B expression in tumor-infiltrating $CD8^+$ T cells (**G**) and NK cells (**H**) in 975A2 tumors 7 days after treatment. Levels of significance for comparison between samples were determined by ANOVA. CTR, vehicle control; DX, doxorubicin; IRI, irinotecan; MTX, mitoxantrone; OXP, oxaliplatin; GZMB, granzyme B. Non-significant data are shown.

Figure S4

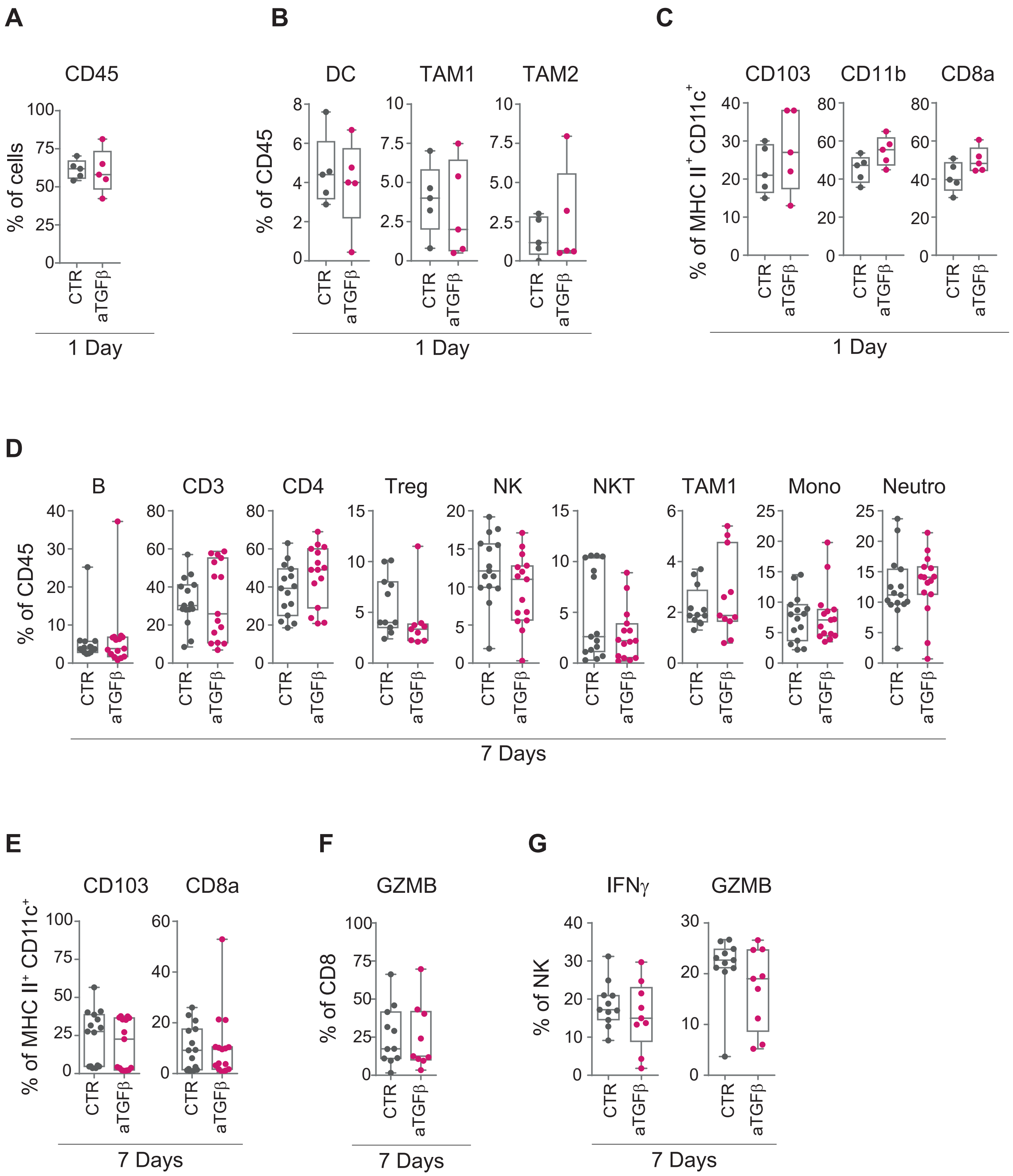

A
CD45
% of cells
CTR
aTGFβ
1 Day
B
DC
TAM1
TAM2
% of CD45
1 Day
C
CD103
CD11b
CD8a
% of MHC II+ CD11c+
1 Day
D
B
CD3
CD4
Treg
NK
NKT
TAM1
Mono
Neutro
% of CD45
7 Days
E
CD103
CD8a
% of MHC II+ CD11c+
7 Days
F
GZMB
% of CD8
7 Days
G
IFNγ
GZMB
% of NK
7 Days

Figure S4 related to Figure 3. Effect of anti-TGFβ treatment on immune content of 975A2 tumors. (**A-E**) Flow-cytometry analysis of the immune content in 975A2 tumors 1 day (**A-C**) and 7 days (**D-E**) after the start of treatment. (**F-G**) Flow-cytometry analysis of the activation status of tumor-infiltrating $CD8^{+}$ T cells (**F**) and NK cells (**G**) in 975A2 tumors 7 days after the start of treatment. Levels of significance for comparison between samples were determined by two-tailed Student's t test. CTR, vehicle control; aTGFβ, anti-TGFβ; GZMB, granzyme B. Non-significant data are shown.

Figure S5

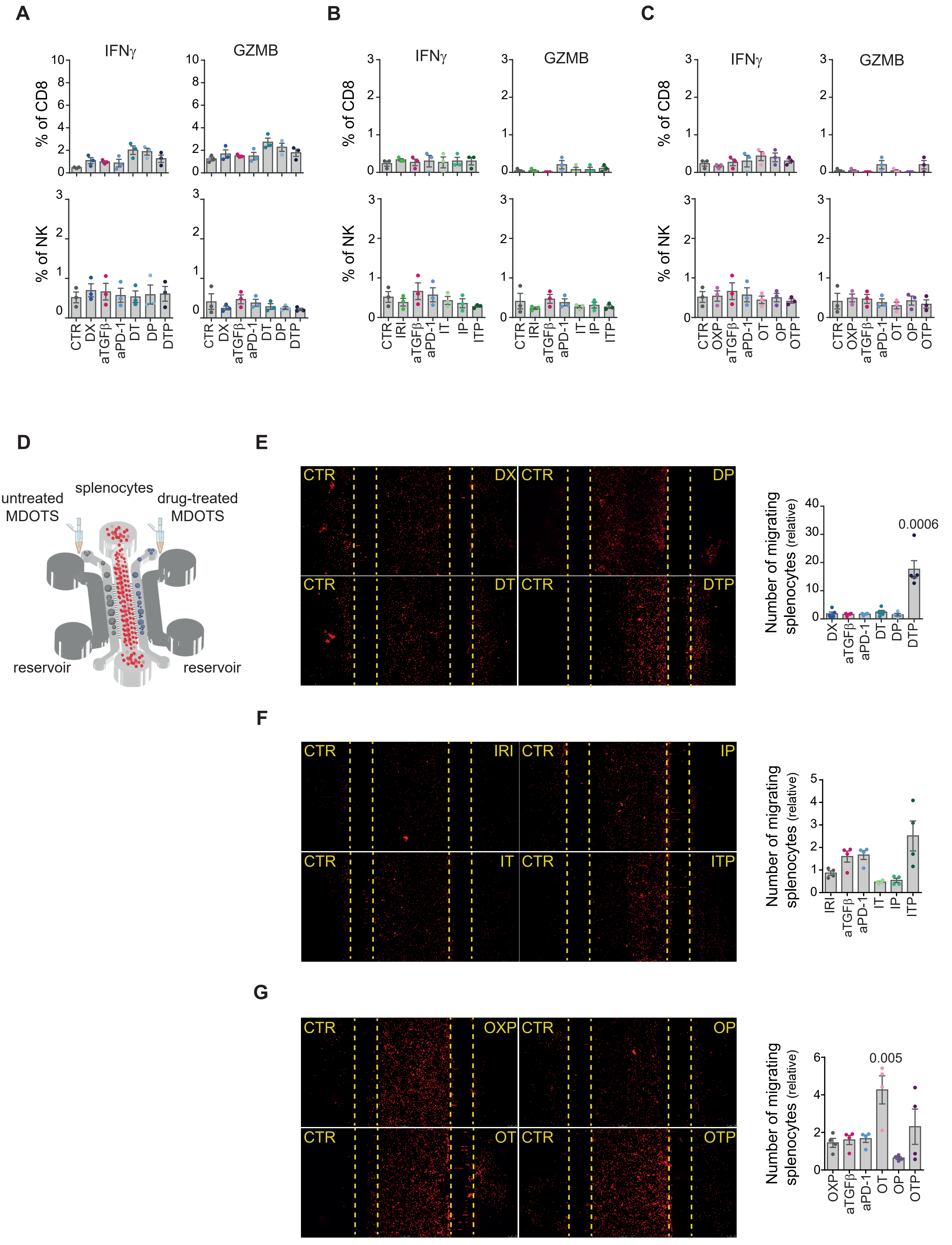
A
B
C
IFNγ
GZMB
% of CD8
% of NK
CTR
DX
aTGFβ
aPD-1
DT
DP
DTP
IRI
IT
IP
ITP
OXP
OT
OP
OTP
D
splenocytes
untreated MDOTS
drug-treated MDOTS
reservoir
reservoir
E
F
G
Number of migrating splenocytes (relative)
0.0006
0.005

Figure S5 related to Figure 4. Effect of combined drug treatment on the recall and activation of CD8$^+$ T cells and NK cells. (**A-C**) Flow-cytometry analyses of IFNγ and granzyme B expression of CD8$^+$ T cells and NK cells from splenocytes co-cultured 24 hours with drug-treated 975A2 MDOTS. (**D**) Schematic representation of the microfluidic device. (**E-G**) Representative images of red-labeled splenocyte migration in microfluidic devices versus drug-treated and untreated 975A2 MDOTS after 24 hours of co-culture. The number of splenocytes from tumor-bearing mice migrating versus treated and untreated 975A2 MDOTS was assessed by ImageJ software. Data are shown as fold change ± SD. Levels of significance were determined by ANOVA. CTR, vehicle control; DX, doxorubicin; IRI, irinotecan; OXP, oxaliplatin, aTGFβ, anti-TGFβ; aPD-1, anti-PD-1; DT, doxorubicin and anti-TGFβ; DP, doxorubicin and anti-PD-1; DTP, doxorubicin, anti-TGFβ and anti-PD-1. IT, irinotecan and anti-TGFβ; IP, irinotecan and anti-PD-1; ITP, irinotecan, anti-TGFβ and anti-PD-1. OT, oxaliplatin and anti-TGFβ; OP, oxaliplatin and anti-PD-1; OTP, oxaliplatin, anti-TGFβ and anti-PD-1; GZMB, granzyme B. Statistically significant P values are shown.

Figure S6

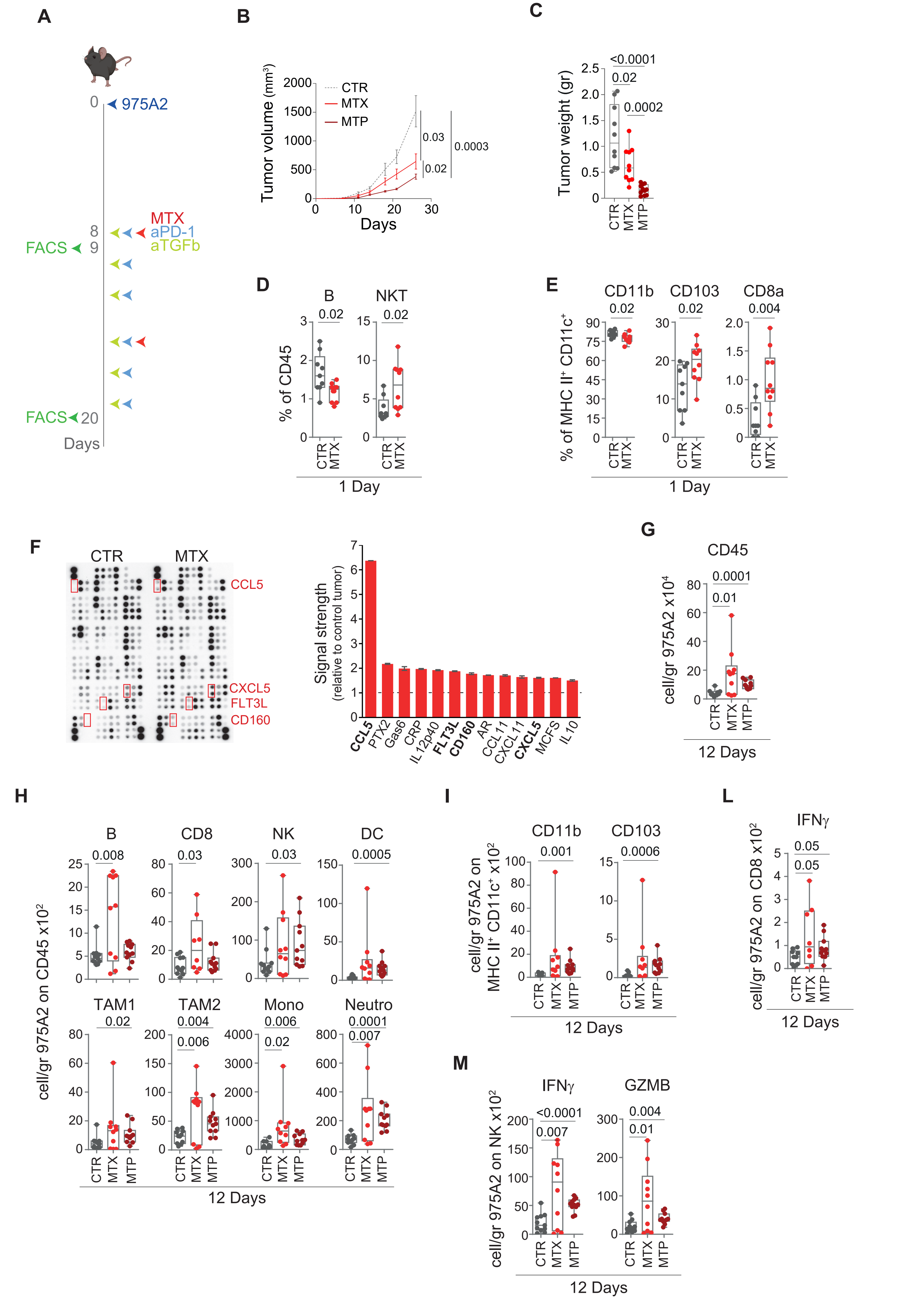

A
0
975A2
8
9
MTX
aPD-1
aTGFb
FACS
FACS
20
Days
B
Tumor volume (mm³)
2000
1500
1000
500
0
CTR
MTX
MTP
0.03
0.02
0.0003
0
10
20
30
Days
C
Tumor weight (gr)
<0.0001
0.02
0.0002
CTR
MTX
MTP
D
B
NKT
% of CD45
0.02
0.02
CTR
MTX
1 Day
E
CD11b
CD103
CD8a
% of MHC II+ CD11c+
0.02
0.02
0.004
CTR
MTX
1 Day
F
CTR
MTX
CCL5
CXCL5
FLT3L
CD160
Signal strength
(relative to control tumor)
CCL5
PTX2
Gas6
CRP
IL12p40
FLT3L
CD160
AR
CCL11
CXCL11
CXCL5
MCFS
IL10
G
CD45
cell/gr 975A2 x10⁴
0.0001
0.01
CTR
MTX
MTP
12 Days
H
cell/gr 975A2 on CD45 x10²
B
0.008
CD8
0.03
NK
0.03
DC
0.0005
TAM1
0.02
TAM2
0.004
0.006
Mono
0.006
0.02
Neutro
0.0001
0.007
CTR
MTX
MTP
12 Days
I
cell/gr 975A2 on MHC II+ CD11c+ x10²
CD11b
0.001
CD103
0.0006
CTR
MTX
MTP
12 Days
L
IFNγ
cell/gr 975A2 on CD8 x10²
0.05
0.05
CTR
MTX
MTP
12 Days
M
IFNγ
GZMB
cell/gr 975A2 on NK x10²
<0.0001
0.007
0.004
0.01
CTR
MTX
MTP
12 Days

Figure S6 related to Figure 5. Treatment of mitoxantrone in combination with TGFβ and PD-1 blockade delays growth of subcutaneously transplanted 975A2 tumors and reshapes intratumoral immune infiltrate. (**A**) Schematic representation of the drug treatment and timing of tumor immune infiltrate analysis. (**B**) Tumor growth of 975A2 injected subcutaneously in C57BL/6 mice and treated as indicated. Significance at day 28 (Mann Whitney test). (**C**) Weight of explanted tumors at day 28 after cell inoculation. (**D-E**) Flow-cytometry analysis of the immune compartment in 1 day-MTX-treated 975A2 tumors. Levels of significance for comparison between samples were determined by two-tailed Student's t test. (**F**) Chemokine expression in 1 day-MTX-treated 975A2 tumor lysates by protein array. Relative chemokine expression based on densitometric analysis is shown on the right. (**G-I**) Flow-cytometry analysis of the immune compartment. (**L-M**) Flow-cytometry analysis of the activation status of tumor-infiltrating $CD8^+$ T cells (**L**) and NK cells (**M**) in 975A2 tumors 12 days after treatment is shown. Levels of significance for comparison between samples in **G-M** were determined by ANOVA. CTR, vehicle control; MTX, mitoxantrone; MTP, mitoxantrone, anti-TGFβ and anti-PD-1; GZMB, granzyme B. Statistically significant P values are shown.

Figure S7

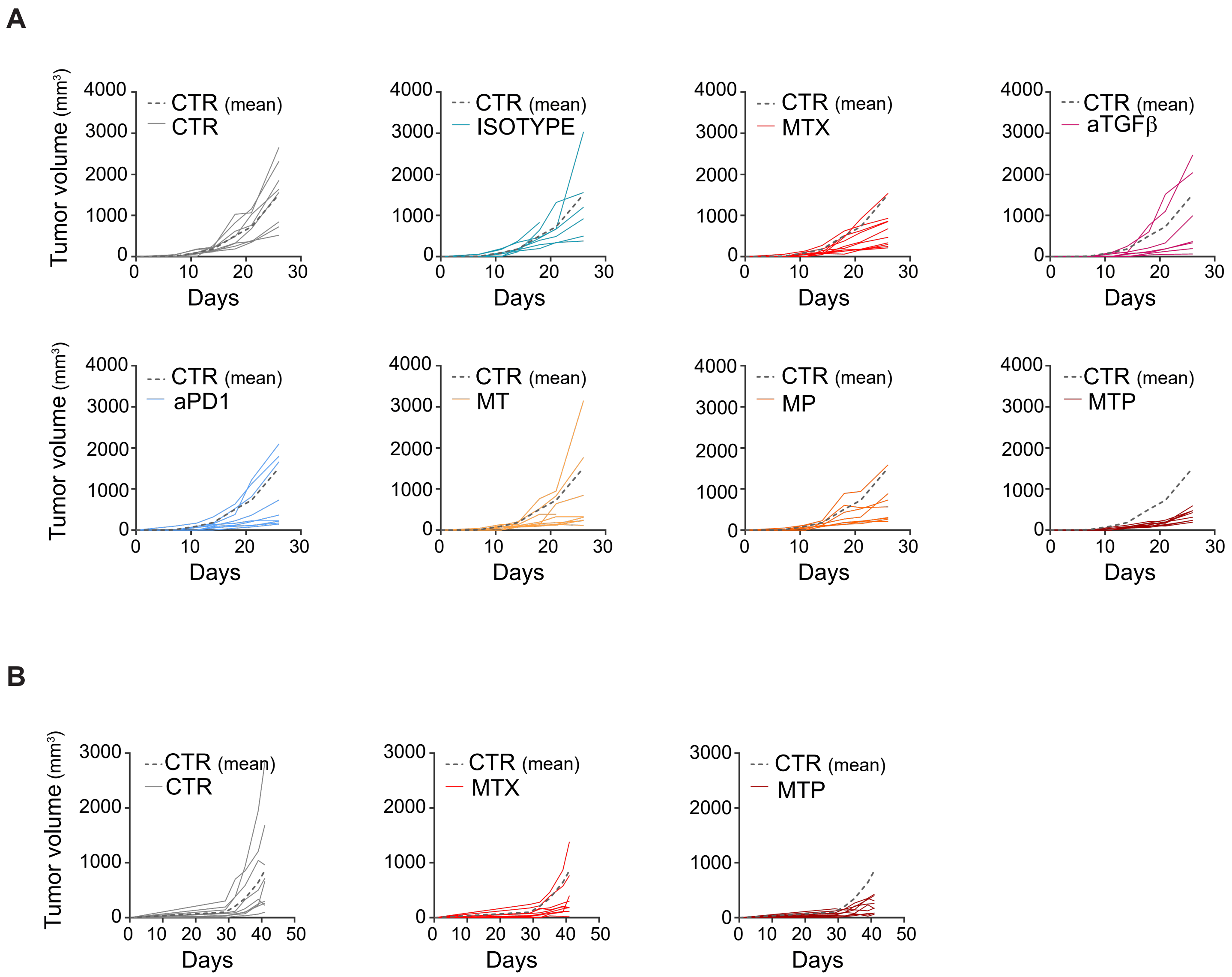

A
Tumor volume (mm³)
4000
3000
2000
1000
0
0 10 20 30
Days
CTR (mean)
CTR
ISOTYPE
MTX
aTGFβ
aPD1
MT
MP
MTP
B
Tumor volume (mm³)
3000
2000
1000
0
0 10 20 30 40 50
Days
CTR (mean)
CTR
MTX
MTP

Figure S7 related to Figure 5. Tumor growth of drug-treated 975A2 and 9464D. Tumor growth of 975A2 (**A**) and 9464D (**B**) injected subcutaneously in C57BL/6 mice and treated as indicated. The average growth of the control tumors is indicated in each graph with a dotted line. CTR, vehicle control; MTX, mitoxantrone; aTGFβ, anti-TGFβ; aPD-1, anti-PD-1; MT, mitoxantrone and anti-TGFβ; MP, mitoxantrone and anti-PD-1; MTP, mitoxantrone. Levels of significance for comparison between samples were determined by Mann Whitney test. Non-significant data are shown.

Figure S8

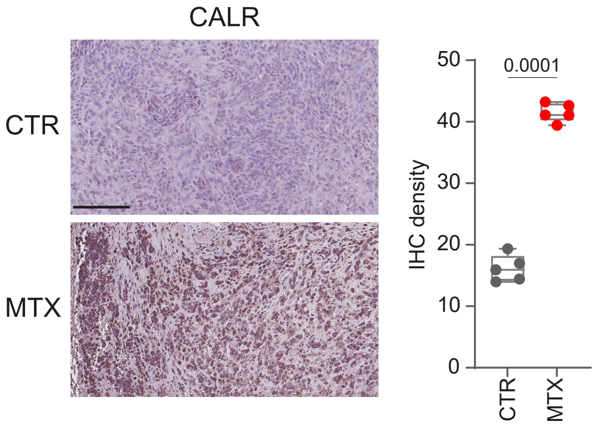

Figure S8 related to Figure 5. Expression of calreticulin in 9464D tumors.

Representative example of calreticulin (CALR) staining in 9464D tumors grown subcutaneously in C57BL/6 mice. Brown, calreticulin-expressing cells. Nuclei were counterstained with hematoxylin (blue). Original magnifications, x20. Scale bars, 30 µm. IHC density was assessed by ImageJ software by measuring 5 different areas of the slide on five independent mice for each condition, and the mean ± SD of integrated density are reported in the histogram. Levels of significance for comparison between samples were determined by two-tailed Student's t test.

Figure S9

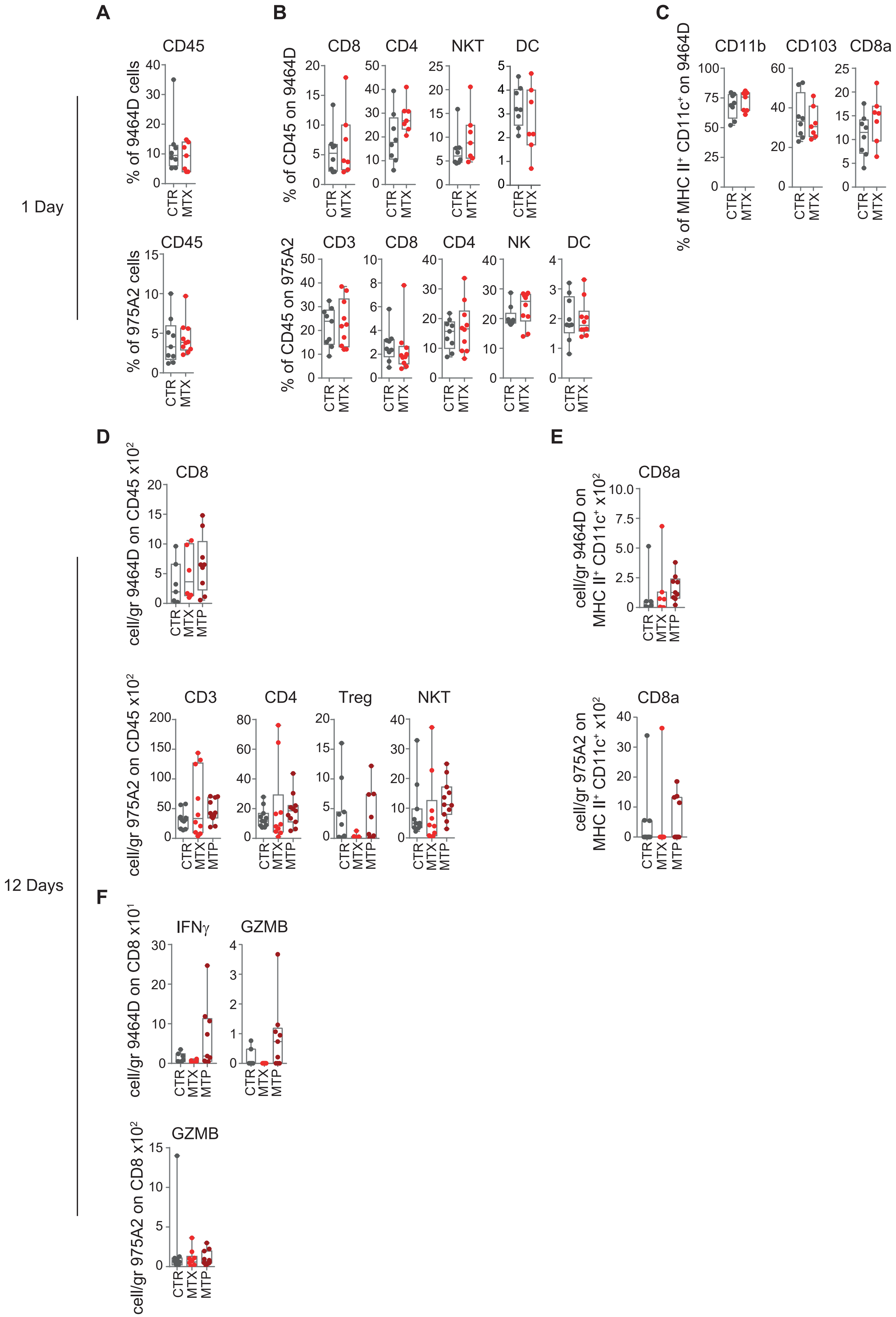
A
B
C
1 Day
CD45
% of 9464D cells
CTR
MTX
CD8
CD4
NKT
DC
% of CD45 on 9464D
CD11b
CD103
CD8a
% of MHC II⁺ CD11c⁺ on 9464D
CD45
% of 975A2 cells
CD3
CD8
CD4
NK
DC
% of CD45 on 975A2
D
E
12 Days
CD8
cell/gr 9464D on CD45 x10²
MTP
CD8a
cell/gr 9464D on MHC II⁺ CD11c⁺ x10²
CD3
CD4
Treg
NKT
cell/gr 975A2 on CD45 x10²
CD8a
cell/gr 975A2 on MHC II⁺ CD11c⁺ x10²
F
IFNγ
GZMB
cell/gr 9464D on CD8 x10¹
GZMB
cell/gr 975A2 on CD8 x10²

Figure S9 related to Figure 5 and Figure S6. Immune content of drug-treated 9464D and 975A2 tumors. Flow-cytometry analysis of the immune content in 9464D and 975A2 tumors (upper and lower panels, respectively) 1 day (**A-C**) and 12 days (**D-E**) after treatment with the indicated drugs. (**F**) Flow-cytometry analysis of the activation status of tumor-infiltrating $CD8^+$ T cells in 9464D and 975A2 tumors (upper and lower panels, respectively) 12 days after treatment. Levels of significance for comparison between samples were determined by two-tailed Student's t test (**A-C**) and ANOVA (**D-F**). CTR, vehicle control; MTX, mitoxantrone; MTP, mitoxantrone, anti-TGFβ and anti-PD-1; GZMB, granzyme B. Non-significant data are shown.

Figure S10

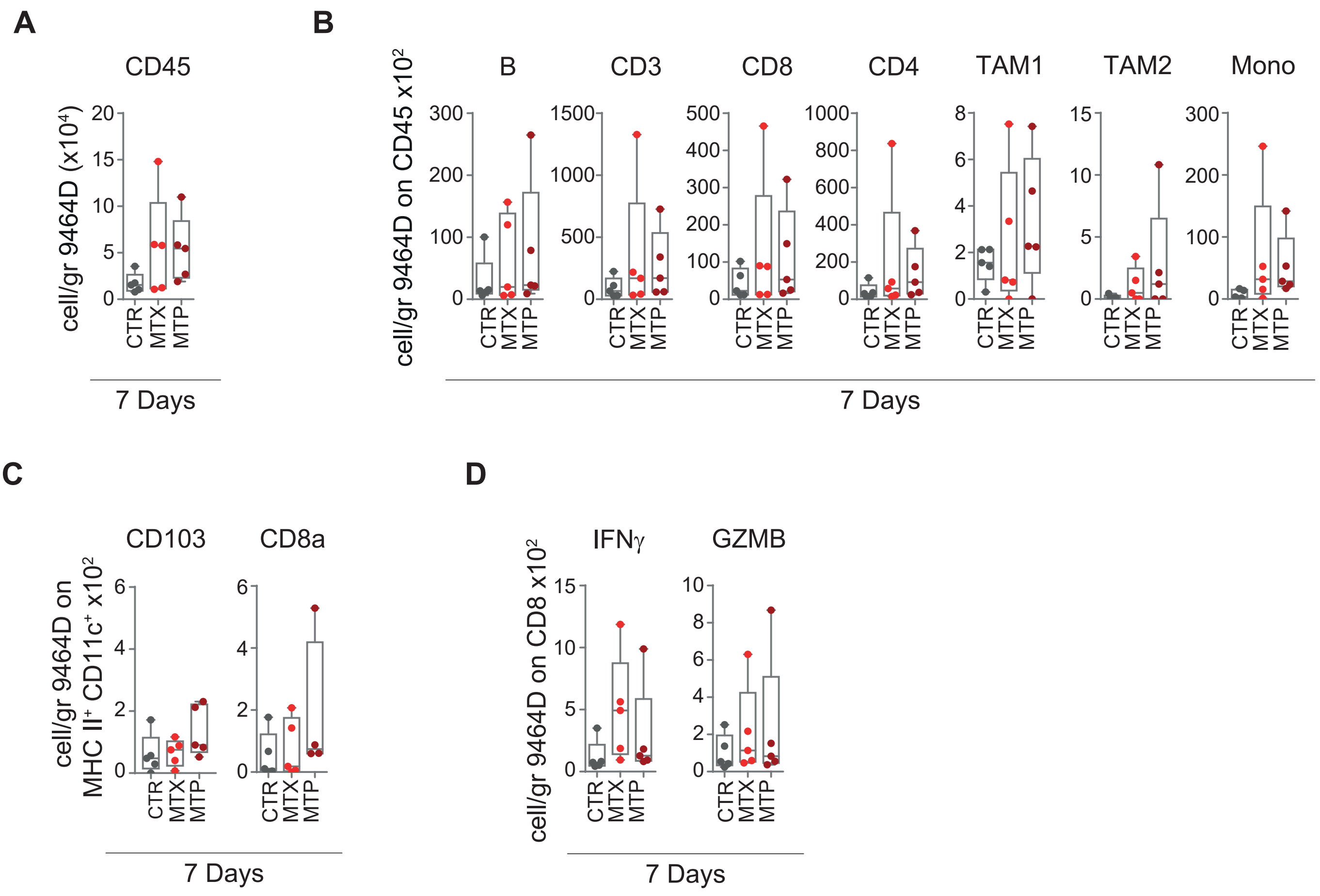
A
CD45
cell/gr 9464D (x10⁴)
20
15
10
5
0
CTR
MTX
MTP
7 Days
B
cell/gr 9464D on CD45 x10²
B
CD3
CD8
CD4
TAM1
TAM2
Mono
300
200
100
1500
1000
500
400
1000
800
600
8
6
4
2
15
10
5
CTR
MTX
MTP
7 Days
C
CD103
CD8a
cell/gr 9464D on MHC II⁺ CD11c⁺ x10²
6
4
2
0
CTR
MTX
MTP
7 Days
D
IFNγ
GZMB
cell/gr 9464D on CD8 x10²
15
10
5
0
8
6
4
2
CTR
MTX
MTP
7 Days

Figure S10 relative to Figure 6. Immune content of drug-treated 9464D grown in the adrenal gland. (**A-C**) Flow-cytometry analysis of the immune content in 9464D 7 days after the start of treatment with the indicated drugs is shown. (**D**) Flow-cytometry analysis of the activation status of tumor-infiltrating $CD8^+$ T cells in 9464D tumors 7 days after the start of treatment is shown. Levels of significance for comparison between samples were determined by ANOVA. Non-significant data are shown. CTR, vehicle control; MTX, mitoxantrone; MTP, mitoxantrone, anti-TGFβ and anti-PD-1; GZMB, granzyme B.

Figure S11

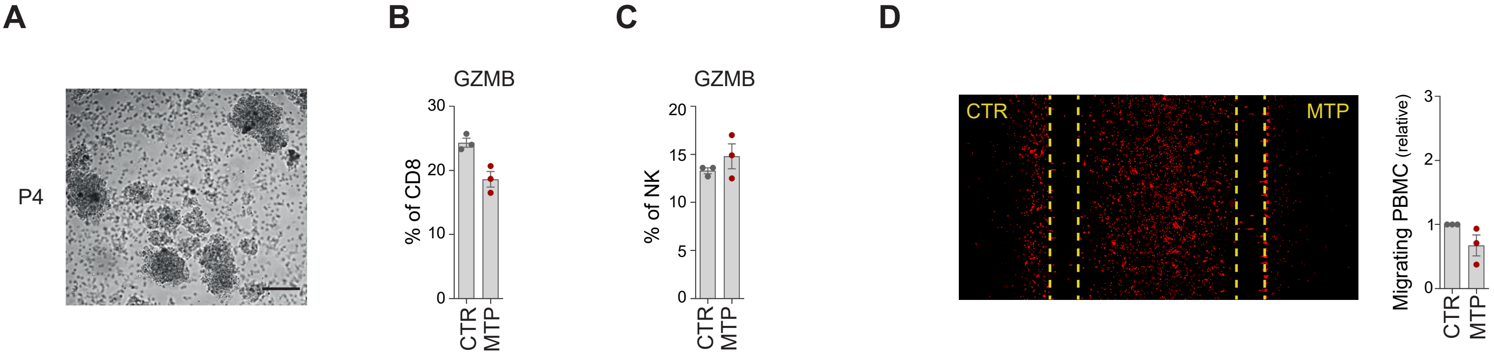
A
P4
B
GZMB
% of CD8
30
20
10
0
CTR
MTP
C
GZMB
% of NK
20
15
10
5
0
CTR
MTP
D
CTR
MTP
Migrating PBMC (relative)
3
2
1
0
CTR
MTP

Figure S11 relative to Figure 7. Mitoxantrone recalls activated $CD8^+$ T cells and NK cells in some PDOTS when combined with TGFβ and PD-1 blockade.

(**A**) Representative images of PDOTS derived from P4 NB patient. Original magnification, x20. Scale bar, 30 µm. (**B and C**) Flow-cytometry analysis of granzyme B expression by $CD8^+$ T cells (**B**) and NK cells (**C**) of autologous PBMCs co-cultured for 24 hours with drug-treated PDOTS. (**D**) Representative images of the migration of red-labeled autologous PBMCs in microfluidic devices to drug-treated and untreated PDOTS after 24 hours of co-culture. The number of PBMCs migrating versus drug-treated and untreated PDOTS was assessed by ImageJ software. Data are shown as fold change ± SD. Levels of significance for comparison between samples were determined by two-tailed Student's t test. MTX, mitoxantrone; CTR, vehicle control; MTP, mitoxantrone, anti-TGFβ and anti-PD-1; GZMB, granzyme B. Non-significant data are shown.

Figure S12

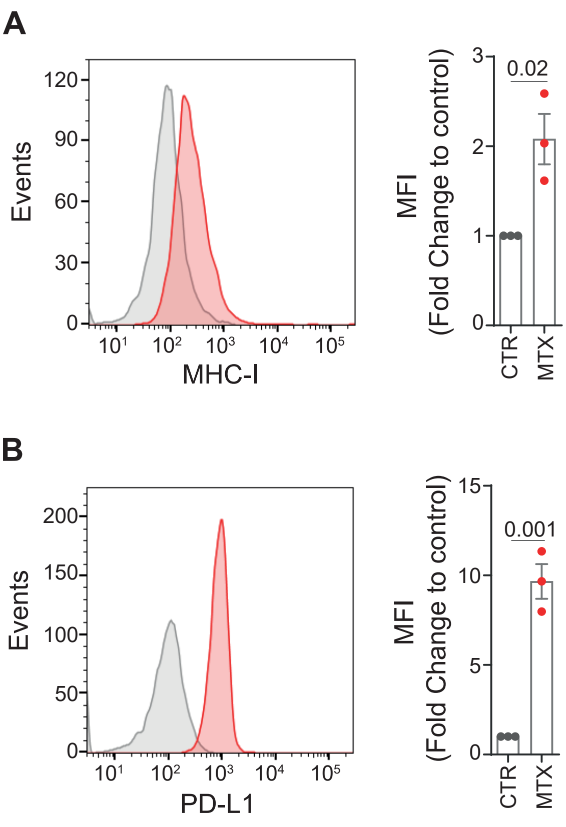
A
120
90
60
30
0
Events
10^1 10^2 10^3 10^4 10^5
MHC-I
3
2
1
0
0.02
MFI
(Fold Change to control)
CTR
MTX
B
200
150
100
50
0
Events
10^1 10^2 10^3 10^4 10^5
PD-L1
15
10
5
0
0.001
MFI
(Fold Change to control)
CTR
MTX

Figure S12. Mitoxantrone TME of drug-treated 9464D grown in the adrenal gland. (**A-B**) Representative flow-cytometry analyses (left) of MHC class I (**A**) and PD-L1 (**B**) in MDOTS derived from 9464D orthotopic model treated 24 hours with MTX. Mice treated with vehicle or MTX are shown as grey and red plots, respectively. The graphs on the right represent the fold changes in MFI ± SD from three independent experiments. Levels of significance for comparison between samples were determined by the two-tailed Student's *t* test. CTR, vehicle control; MTX, mitoxantrone; MFI, mean fluorescence intensity. Statistically significant, *P* values are shown.

Table S1. List of antibodies

MARKER	Fluorochrome	Clone	Company	Catalog	Application	Diluition
anti-mouse CD45	BV605	30-F11	BD Biosciences	563053	FACS	1:40
anti-mouse B220	APC	RA3-6B2	BD Biosciences	553092	FACS	1:40
anti-mouse CD3	BUV395	145-2C11	BD Biosciences	565992	FACS	1:40
anti-mouse CD4	PE CY7	GK1.5	BD Biosciences	563933	FACS	1:40
anti-mouse CD8	BUV805	53-6.7	BD Biosciences	612898	FACS	1:40
anti-mouse CD25	PE	PC61	BD Biosciences	553866	FACS	1:40
anti-mouse NK1.1	BV421	PK136	BD Biosciences	562921	FACS	1:40
anti-mouse FOXP3	PerCP CY5.5	FJK-16s	eBiosciences	45-5773-82	FACS	1:10
anti-mouse CD44	BV421	IM7	BD Biosciences	563970	FACS	1:40
anti-mouse CD62L	APC	MEL-14	BD Biosciences	561919	FACS	1:40
anti-mouse CD69	APC CY7	H1.2F3	BD Biosciences	561240	FACS	1:40
anti-mouse TGFβ	PE	/	R&D SYSTEMS	IC1835P	FACS	1:10
anti-mouse CD11B	PerCP CY5.5	M1/70	BD Biosciences	550993	FACS	1:40
anti-mouse CD11C	BUV395	N418	BD Biosciences	744180	FACS	1:40
anti-mouse MHC II	APC	M5/114.15.2	BioLegend	107614	FACS	1:40
anti-mouse Ly6C	APC CY7	HK1.4	BioLegend	128026	FACS	1:40
anti-mouse Ly6G	PE CY7	1A8	BioLegend	127618	FACS	1:40
anti-mouse F4/80	PE	BM8	BioLegend	123109	FACS	1:40
anti-mouse CD103	BV421	2E7	BioLegend	121421	FACS	1:40
anti-mouse CD40	PE	1C10	eBiosciences	12-0401-82	FACS	1:40
anti-mouse CD86	PE CY7	GL1	eBiosciences	25-0862-82	FACS	1:40
anti-mouse IFNγ	PE CY7	XMG1.2	BD Biosciences	561040	FACS	1:10
anti-mouse GRZB	APC	NGZB	eBiosciences	17-8898-82	FACS	1:10
anti-mouse PD-1	APC	J43	BD Biosciences	562671	FACS	1:40
anti-mouse PD-L1	BV711	MIH5	BD Biosciences	563369	FACS	1:40
anti-mouse MHC-I	PerCP Cy5.5	C3H	Biolegend	114619	FACS	1:40
anti-mouse GD2	AF647	14.G2a	BD Biosciences	562096	FACS	1:40
anti-human CD45	BUV805	HI30	BD Biosciences	612891	FACS	1:40
anti-human CD3	BB700	SK7	BD Biosciences	566575	FACS	1:40
anti-human CD8	BUV395	RPA-T8	BD Biosciences	563795	FACS	1:40
anti-human NKp46	BV711	9E2/NKp46	BD Biosciences	563043	FACS	1:40
anti-human GZMB	BV510	GB11	BD Biosciences	563388	FACS	1:40
anti-mouse/human synaptophysin	/	SY38	Invitrogen	MA1-213	IHC	1:50
calreticulin	/	/	Abcam	ab2907	IHC	1:500
anti-mouse NK.1.1	/	PK136	Invitrogen	MA1-70100	IF	1:50
anti-mouse GZMB	/	D2H2F	Cell Signaling	17215	IF	1:100
Goat-anti−mIgG	Alexa-Fluor488	/	Invitrogen	A-11017	IF	1:500
Goat-anti-rIgG	Alexa-Fluor594	/	Invitrogen	A-11037	IF	1:500

Table S2: Clinical and genetic characteristics of 7 newly diagnosed NB patients.

Patient	Age ad diagnosis (months)	Gender	Stage (INRG)	Histologic classification (INCP or Shimada)	Grade of NB differentiation (INCP or Shimada)	MYCN status	PDOTS formation	PDOTS responsiveness
P1	3	Male	L1	Favorable	Undifferentiated	Not amplified	No	/
P2	18	Male	L1	Intermediate	Undifferentiated	Not amplified	No	/
P3	13	Male	L2	Favorable	Undifferentiated	Not amplified	No	/
**P4**	35	Female	L2	Unfavorable	Undifferentiated	Not amplified	Yes	No
**P5**	22	Female	M	Unfavorable	Undifferentiated	Not amplified	Yes	Yes
**P6**	96	Male	M	Unfavorable	Undifferentiated	Not amplified	Yes	Yes
**P7**	1	Male	MS	Favorable	Undifferentiated	Not amplified	Yes	Yes

Patients analyzed are indicated in bold.
